# Decoding Structural Properties of a Partially Unfolded Protein Substrate: En Route to Chaperone Binding

**DOI:** 10.1371/journal.pcbi.1004496

**Published:** 2015-09-22

**Authors:** Suhani Nagpal, Satyam Tiwari, Koyeli Mapa, Lipi Thukral

**Affiliations:** 1 CSIR-Institute of Genomics and Integrative Biology, New Delhi, India; 2 Academy of Scientific and Innovative Research (AcSIR), New Delhi, India; 3 Department of Life Sciences, School of Natural Sciences, Shiv Nadar University, Dadri, Uttar Pradesh, India; Weizmann, ISRAEL

## Abstract

Many proteins comprising of complex topologies require molecular chaperones to achieve their unique three-dimensional folded structure. The *E.coli* chaperone, GroEL binds with a large number of unfolded and partially folded proteins, to facilitate proper folding and prevent misfolding and aggregation. Although the major structural components of GroEL are well defined, scaffolds of the non-native substrates that determine chaperone-mediated folding have been difficult to recognize. Here we performed all-atomistic and replica-exchange molecular dynamics simulations to dissect non-native ensemble of an obligate GroEL folder, DapA. Thermodynamics analyses of unfolding simulations revealed populated intermediates with distinct structural characteristics. We found that surface exposed hydrophobic patches are significantly increased, primarily contributed from native and non-native *β*-sheet elements. We validate the structural properties of these conformers using experimental data, including circular dichroism (CD), 1-anilinonaphthalene-8-sulfonic acid (ANS) binding measurements and previously reported hydrogen-deutrium exchange coupled to mass spectrometry (HDX-MS). Further, we constructed network graphs to elucidate long-range intra-protein connectivity of native and intermediate topologies, demonstrating regions that serve as central “hubs”. Overall, our results implicate that genomic variations (or mutations) in the distinct regions of protein structures might disrupt these topological signatures disabling chaperone-mediated folding, leading to formation of aggregates.

## Author Summary

Several non-native proteins require molecular chaperones for proper folding. Many unfolded proteins if not folded accurately, become causal factors in various types of misfolding or aggregation induced diseases such as Alzheimer′s, Huntington′s and several other neurodegenerative disorders. However, structural information of non-folded proteins especially chaperone-dependent proteins is difficult to probe experimentally due to their inherent aggregation propensities. In this work, we study DapA protein, which exhibits obligate requirement on GroEL chaperonin machinery for its folding. We use molecular dynamics simulations to reveal populated intermediate structures of DapA in atomic details. The most plausible intermediate was found to be in agreement with recently reported hydrogen-exchange experimental data. Significant increase in surface exposed hydrophobicity was observed in intermediates compared to native, which was further validated using ANS binding experiments. We also constructed network model of these intermediates that provides remarkable insights into stable hubs (or important residues) underlying diverse states of unfolded proteins. In summary, our work provides a molecular picture of an unfolded protein that is en route to chaperone binding, and these underlying structural properties might act as a molecular signal for their productive folding.

## Introduction

Properly folded proteins is a prerequisite in nearly every cellular process. Several proteins fold spontaneously while others rely on molecular chaperones to reach their native states [1]. GroEL, an indispensable chaperone of *Escherichia coli*, selectively binds with unfolded proteins to assist in their productive folding [2–6]. Although 250 cytosolic proteins interact with GroEL, ≈ 57 substrates have an obligate dependence on GroEL for folding [7, 8]. The most prominent feature associated with GroEL dependence is (*α*/*β*)_8_ TIM (triosephosphate isomerase) barrel fold, albeit, it is also possessed by many cellular proteins which are GroEL independent folders [7, 9]. Thus, specific proteins lacking native tertiary structure [10], seem to be a universal precursor for GroEL-substrate binding. These considerations underlie an important unanswered question: What are the structural characteristics of these non-native substrates, that is, do they have some feature(s) that may be unique and as a consequence more prone for chaperone targeting?

Since GroEL binds to kinetically-trapped intermediates [11, 12], it is likely that a combination of sequence and structural motifs accessible on these intermediates would act as driving forces for GroEL interaction. Based on sequence-based features, Horovitz and colleagues have identified that GroEL interacting proteins have low folding propensities and high translational efficiencies compared to other *E.coli* proteins [13]. However, structural characterization of folding intermediates using current experimental techniques is extremely challenging and the problem is further exacerbated due to their inherent aggregation propensities. Due to this complexity, previous investigations on GroEL-bound proteins report diverse structural features, including *α*-helical content [14, 15], exposed *β*-sheet [16–18], or random coil conformations devoid of any stable native-like tertiary contacts [19, 20]. The presence of hydrophobic surfaces has been identified as a critical factor for GroEL binding, but structural composition of these surfaces are difficult to observe experimentally [4, 11, 21–23]. Nevertheless, other unknown structural features are likely to play an additional modulatory role that should favor chaperone binding.

**Fig 1:**
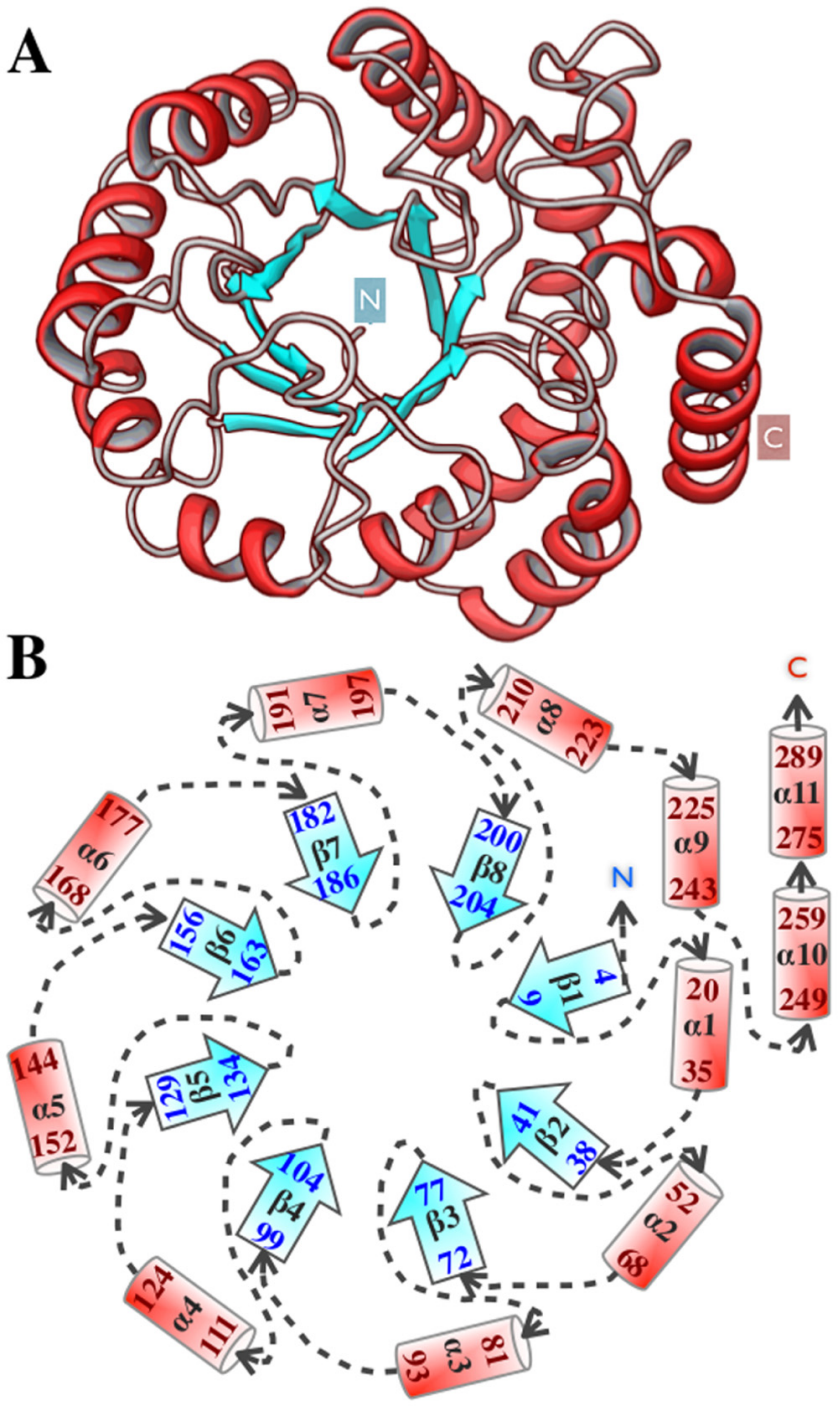
DapA structure. A) The protein is composed of eleven *α*-helices surrounding the *β*-barrel superstructure composed of eight parallel *β*-strands shown in red and blue, respectively. B) Schematic representation of DapA topology displaying (*α/β*)_8_ TIM barrel fold along with C-terminal *α*-helices (*α*9–11). The terminal residues of each secondary structural elements are labeled.

In our earlier work, using an array of cellular substrates of GroEL, we have shown that not only the primary sequence of substrates but also the structural features such as surface hydrophobicity, extent and type of secondary structure of non-native refolding intermediates dictate chaperone targeting [24]. In this study, we aim to decipher the specific features governing non-native state of a GroEL substrate, DapA (Dihydrodipicolinate synthase). DapA is an essential tetrameric enzyme and is 292-residue long protein belonging to the class IV family of GroEL substrates, and is known to be an obligate chaperone substrate [3, 7]. Fig 1A shows the DapA monomer structure consisting of a TIM barrel domain and C-terminal domain constituting of three *α*-helices. This fold type exhibits a complex topology consisting of eight *α*-helices in the periphery and eight *β*-strands in the core as shown schematically in Fig 1B. One of the most intriguing features among members of these TIM-barrel fold substrates is that although they comprise of the same tertiary fold, the sequence similarity is less than 20%.

In this study we provide a comprehensive network map of the intermediates of an obligate GroEL substrate, DapA. For this, we combined classical and replica-exchange molecular dynamics simulations to extract stable intermediates. We validated the structural properties of these conformers using experimental data, including CD and ANS binding, and previously reported hydrogen-exchange experiments of DapA. Further, control simulations of GroEL independent folder [25], Triosephosphate isomerase (TIM) shows dramatic difference in unfolding behavior compared to DapA. Our study (i) provides network description of crucial but poorly characterized non-native substrate of chaperone, (ii) structural characterization of these intermediates reveal their compact and distinct topology, and (iii) our assessment of dynamical properties demonstrates that there exists an underlying network highlighting intra-protein communication pathways. The signatures derived from these networks are crucial to understand physico-chemical properties of a large number of proteins that achieve native state through chaperone-mediated folding.

## Materials and Methods

### Molecular Dynamics simulations

The MD simulations were performed using the program GROMACS [26], and the OPLS all-atom force field [27] was used for the protein. The water molecules were modeled with the TIP3P representation [28]. Periodic boundary conditions were used and long-range electrostatic interactions were treated with the Particle Mesh Ewald (PME) summation using grid spacing of 0.16 nm combined with a fourth-order cubic interpolation to deduce the potential and forces in-between grid points [29]. The real space cutoff distance was set to 1.0 nm and the van der Waals cutoff to 1.0 nm. The bond lengths were fixed [30] and a time step of 2 fs for numerical integration of the equations of motion was used. Coordinates were saved every 10 ps.

The simulations were performed using the crystal structure of DapA taken from PDB database (ID: 1DHP) as the starting structure. Three independent MD trajectories, each 3 *μ*s long at 310 K, 360 K and 400 K were carried out. The combined time-scale of our simulations is 27 *μ*s. The protein was placed in a dodecahedral water box (volume = 325.13 nm^3^) large enough to contain protein and at least 1.0 nm of solvent on all sides. The structure was solvated with 9,864 water molecules, and five Na+ ions were added to neutralize the system. The total number of atoms for DapA were 32,834. The starting structure was subjected to energy minimization using the steepest descent method. For each temperature (310 K, 360 K and 400 K), simulations were subjected to Nose-Hoover T-coupling bath to maintain the exact temperature [31]. The structures were then subjected to Berendsen barostat for pressure coupling at 1 bar [32], before the 3 *μ*s production run was started.

In addition to DapA simulations, control simulations of TIM protein were also performed. The crystal structure of the protein was taken from PDB Database (ID:4IOT) as the starting structure. Two independent MD simulations, each 3 *μ*s long at 400 K were done. The structure was solvated with 8,650 water molecules and seven Na+ ions were added to neutralize the system. Total number of atoms in the entire system were 30,192. The structure was subjected to similar MD protocol as described above, before the final production run was started.

### Replica Exchange Molecular Dynamics

In addition to the constant temperature MD simulations (CTMD), Replica Exchange Molecular Dynamics (REMD) of DapA protein were also performed [33] as a tool for validation. It comprised of 96 replicas with temperature ranging from 280 K to 426 K. The initial estimate of temperature range was generated through REMD temperature generator program [34]. Preliminary REMD for 40 ns/replica was performed to calculate initial estimate of temperature range and the potential energy of the system. Thus, these initial REMD trajectories were used for estimating the initial temperature spacing based on the polynomial fit [35]. The exchange probability calculated was 20% to 40% for an exchange to take place between the neighboring replicas, and state exchange attempted between all replicas is 4 ps. Each trajectory was simulated for 700 ns with a partially folded structure (RMSD ≈ 0.48 nm) as the starting structure using the above mentioned protocol. The unfolded fraction from RMSD values of all 96 replicas were calculated for each temperature with the cutoff 0.3–0.9 nm as the unfolded state. The set-up of REMD was performed using the NPT ensemble similar to MD protocol as explained above.

### Analysis of the trajectories

Free Energy Contour Maps of DapA: The maps were determined by calculating the normalised probability distribution based on a set of order parameters. We define the free energy based on probability distribution using the following expression:

$$\Delta A_{ref\to i}=-RT\ln\frac{p_{i}}{p_{ref}}$$ (1)

in this, the probability of going from a reference state, *ref*, of the system to a generic state,*i*, (e.g., from folded to unfolded) at constant temperature and constant volume was evaluated. Where *R* is the ideal gas constant, *T* is the temperature and *p_*i*_* and *p_*ref*_* are the probabilities of finding the system in state *i* and state *ref*, respectively. We describe the free energy maps as a function of two order parameters, namely the root mean square deviation (RMSD), and *ρ* i.e., fraction of native side-chain contacts. The contact between sidechain is formed when the minimum distance between the atoms belonging to the sidechains is ≤ 0.55 nm. Structures sampled every 100 ps were projected onto the RMSD-*ρ* plane. A grid of 20x20 was used to divide this plane in 400 cells and for every cell the number of points was counted and the relative probability was calculated, allowing ΔA_*ref* → *i*_ to be evaluated. The reference state was chosen to be the grid cell with the highest probability, which corresponds to the folded ensemble in the 310 K, I_1_ ensemble at 360 K and I_2_ ensemble at 400 K. We also constructed two additional free energy contour maps (Q-RMSD and SASA-RMSD) for validating the obtained I_2_ ensemble.

Extraction of the intermediate ensembles: Free energy contour map was constructed as a function of two order parameters, namely the root mean square deviation (RMSD) and *ρ* parameter for three trajectories for each temperature. All the structures were projected on this plane. The cut-off values for defining N, I_1_ and I_2_ conformations were chosen based on RMSD-*ρ* free energy contour map: structures populating the folded basin were extracted from 310 K map within defined RMSD (0.16–0.26 nm) and *ρ* (0.79–0.83) values. The 360 K map primarily populated I_1_ structures (0.3–0.42 nm;0.65–0.7) and 400 K was populating I_2_ structures (0.65–0.79 nm;0.422–0.47). The additional free energy contour maps Q-RMSD and SASA-RMSD also populates I_1_ (0.3–0.4 nm;0.59–0.645) (0.3–0.41 nm;70–75 nm^2^) and I_2_ (0.65–0.83;0.44–0.51) (0.65–0.83;72.5–81 nm^2^) structures.

Hydrogen bonding analysis for comparison with HDX-MS experiments: Solvent mediated hydrogen bonds of I_2_ structures were calculated from three 400K trajectories. Each peptide segment (based on experimental data) was taken as a separate group and inter-hydrogen bonds were monitored across the I_2_ trajectories with cut off ≈ 0.35 nm. The average hydrogen bonding of solvent and each peptide across a trajectory has been plotted and the error bars represents data across three trajectories.

Secondary structure probability: Propensity of each residue to form a particular secondary structure based on the DSSP algorithm [36] was calculated based on number of times each residue lies in a specific secondary structural element with respect to native topology. Analysis were carried for every 100 ps frame of I_1_ and I_2_ trajectories.

Hydrophobic patch analysis: The exposed surface hydrophobic patches were calculated using the tool Quilt [37]. It provides atomistic description of surface non-polar atoms that are contributing to a contiguous hydrophobic patch using a dot representation of a solvent accessible surface area. Using the polar expansion radius of 0.14 nm, the area and the corresponding number of atoms constituting the patch(es) are reported. In this work, to extract the exposed surface hydrophobic patches (ESHP), the representative structures for N, I_1_ and I_2_ ensemble were subjected to Quilt protocol. For each conformer, we obtained top five patches ranked according to ESHP, and only patches with surface area ≥ 300 Å^2^ were considered significant for further analysis as shown in S1 Table.

Intra-molecular network and communication pathway: The network and communication pathway for N, I_1_ and I_2_ ensembles were built using MONETA [38]. Similar methodology has been applied previously to construct protein allosteric pathways [38–40]. Briefly, it builds a modular network representation of the protein, composed of interconnected clusters of residues representing communication pathways (Cps). The representation is obtained from topology of the protein and from inter-residue dynamical correlations extracted from MD simulations. In our case, the calculation was done on N, I_1_ and I_2_, ensemble trajectories. The structural features of the protein internal dynamics were identified in each analyzed conformational state (N, I_1_ and I_2_). Communication propensity matrices were computed using cpptraj module of AMBER tools, whereby average smallest distances between each C-*α* residue pairs is calculated and represented in a form of distance matrices. Two residues i and j were considered as neighbors if the average smallest distance between them was lower than a given threshold of 3.6 Å. MONETA uses the concept of communication propensity to characterize the communication pathways (Cps). The communication pathway connects major hubs in the intra-protein network and further be implicated to impart stability to the overall topology of the conformation. Cps are chains of residues with high communication propensities between each node. It is defined as the inverse relation to their commute time CT (i,j), expressed as a function of the variance of the inter-residue distance

$$CT\left(i,j\right)=<{\left(d\left(ij\right)-\delta \left(ij\right)\right)}^{2}>$$ (2)

where,

d(ij)=|r(i)r(j)| (3)

is the distance between the C-*α* atoms of residue i and j. The Cps are grown in an order to ensure that any two adjacent residues are connected by non-covalent interactions and that every residue in the CP is reachable to any other point by a short CT. Non-bonded interactions (interaction matrices) are analysed along the trajectory using LIGPLOT. Two residues were considered as interacting for at least 50% of the frames. The CT threshold was 0.1 for all the studied trajectories. Visualization and graphical analysis were done using GEPHI 0.8.2 and CHIMERA.

### Experimental methods

CD spectroscopy: CD spectra of the protein (final 1 *μ*M) was obtained by a JASCO spectro polarimeter using a cuvette of 1 cm path length at 25°C in buffer A (25 mM Tris-HCl, pH 7.5, 80 mM KCl and 5 mM MgCl_2_. Thermal denaturation of DapA was carried out by increasing the temperature from 25°C to 90°C with a rate of temperature increment of 1°C/minute. CD signals at 222 nm were plotted against temperature to obtain the experimental Tm.

ANS binding of Non-Native intermediates of DapA: Purified DapA protein (final 2 *μ*M) was taken in 25 mM Tris-HCl, pH 7.5, 80 mM KCl, 5 mM MgCl_2_ buffer containing 10 *μ*M (final conc.) 1-anilinonaphthalene-8-sulfonic acid (1–8 ANS). Emission spectra of 1–8 ANS was recorded in a Fluoromax -4 spectroflurometer (Jobin Yvon) after excitation at 365 nm wavelength at 30°C. DapA was thermally denatured by gradually increasing the temperature from 30°C to 60°C with an increment of 5°C at each step followed by 30 min. incubation after each 5°C increment. Emission spectra of 1–8 ANS was recorded after every 5°C increment following 30 minutes of incubation at that temperature.

## Results

### Free-energy Contour Maps of DapA reveal stable intermediates

**Fig 2:**
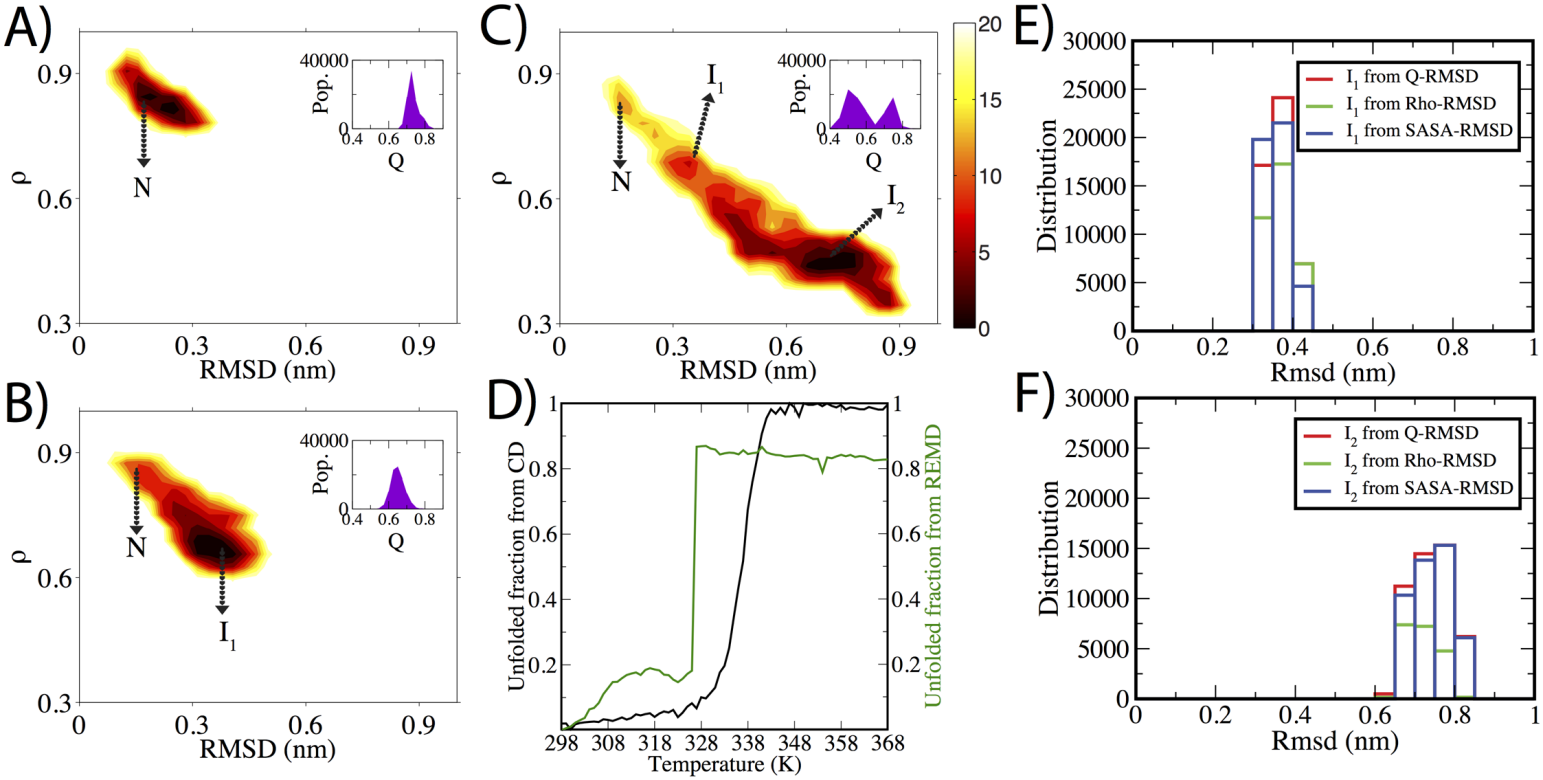
Thermodynamics of DapA unfolding. A-C) Free energy contour maps of DapA as a function of RMSD and *ρ* for three different temperatures, namely; 310 K, 360 K and 400 K, respectively. The color bar denotes the Gibbs free energy in kJ/mol. The inset within the maps show distribution of fraction of native contacts, Q. Q is defined by the total number of native contacts for each trajectory frame divided by the total number of contacts in the native structure. D) Thermal melting curve of DapA derived from CD (in black) and REMD simulations (in green), is depicted (see Methods for details). The molar ellipticity values obtained at 222 nm were normalized between 0 to 1 as a function of temperature. E-F) displays RMSD distributions of *ρ*-RMSD, Q-RMSD SASA-RMSD maps for I_1_ and I_2_ configurations, respectively. The RMSD was calculated with respect to the native structure.

Here, we probed the non-native ensemble of the DapA protein using computer simulations. First, the thermodynamics was quantified using multiple *μ*s-long atomistic MD simulations at various temperatures (310 K, 360 K, and 400 K) starting from the folded native structure (see Methods for details). Several previous theoretical investigations on protein unfolding reactions have also leveraged high-temperature simulations to trace stable unfolded states, and the findings were found to be consistent with various experimental variables [41–48]. The two-dimensional unfolding free energy contour maps as a function of RMSD and fraction of native side-chain contacts, *ρ* are shown in Fig 2A–2C with the inset showing the fraction of native contacts; Q. For physiological temperature, DapA was predominantly in the native state (N), with a small fraction exhibiting a subtle structural change. This was due to outward extension of the C-terminus loop which accounts for flexibility within the native state(Fig 2A). At 360 K, in addition to native basin (RMSD ≈ 0.12–0.21 nm; *ρ* ≈ 0.9–0.8), we also observed the presence of a partially unfolded state, I_1_ with 65–70% of fraction of native contacts (Q) still intact (Fig 2B). However, at 400 K we observed a rather rough free energy surface where the protein traversed from the native structure to I_1_ (Q; 65–80%), and finally progressed towards a highly populated state I_2_ (Q; 40 to 50%), with 9 kJ/mol energy barrier (Fig 2C). Interestingly, I_2_ ensemble encompasses the broad minima and populates structures with *ρ* reduced from ≈ 0.9 nm in the native state to ≈ 0.43 nm in the I_2_ state. Thus, two distinct intermediate ensembles (I_1_ and I_2_) were identified. To probe the sensitivity of intermediate structures derived from selected reaction coordinates, two additional free energy contour maps were calculated as a function of Q-RMSD, and RMSD-SASA pair of variables (S1 Fig). Fig 2E and 2F show the overlap between three maps for I_1_ and I_2_ configurations, which depict excellent agreement and further supports the intermediate ensembles. Therefore, for further analysis we extracted N, I_1_ and I_2_ population from the original free energy planes as described in the Methods.

We also report Replica-Exchange Molecular Dynamics (REMD) simulations [33], an approach applied to several protein folding studies [49, 50], whereby multiple copies or replicas of system are simulated in parallel at different temperatures, and configurations are periodically exchanged between two replicas in a manner that preserves detailed balance. REMD simulations are often used to produce melting curves, showing fraction of folded or native structures as a function of temperatures. In addition, we performed Circular Dichroism (CD) spectroscopy experiments to investigate thermal stability of DapA. Fig 2D shows the melting curve obtained from the simulations and CD measurements, and the derived T_*m*_ from the CD spectra is 333.15 K, compared to 326.15 K estimated from REMD simulations. In addition, the stable intermediate state (I_2_) share common structural features in both simulations as shown in S2 Fig. The agreement with the REMD simulations is invigorating, and provides more reliability to our proposed intermediate structures.

We further examined the statistical reliability of simulations. Firstly, the convergence analysis of constant temperature and replica-exchange simulations is shown in S3 Fig. Several events contributing to I_2_ transitions in both (constant-temperature and replica-exchange) simulations reach conformational stability across multiple trajectories. Secondly, we compared REMD simulations with the constant-temperature simulations. Although we observed significant decrease in intra-molecular hydrogen-bond network as a function of temperature in REMD (S4 Fig), corresponding CTMD trajectory shows more conformational heterogeneity.

### Validation of intermediate structures with recent HDX-MS experiments

**Fig 3:**
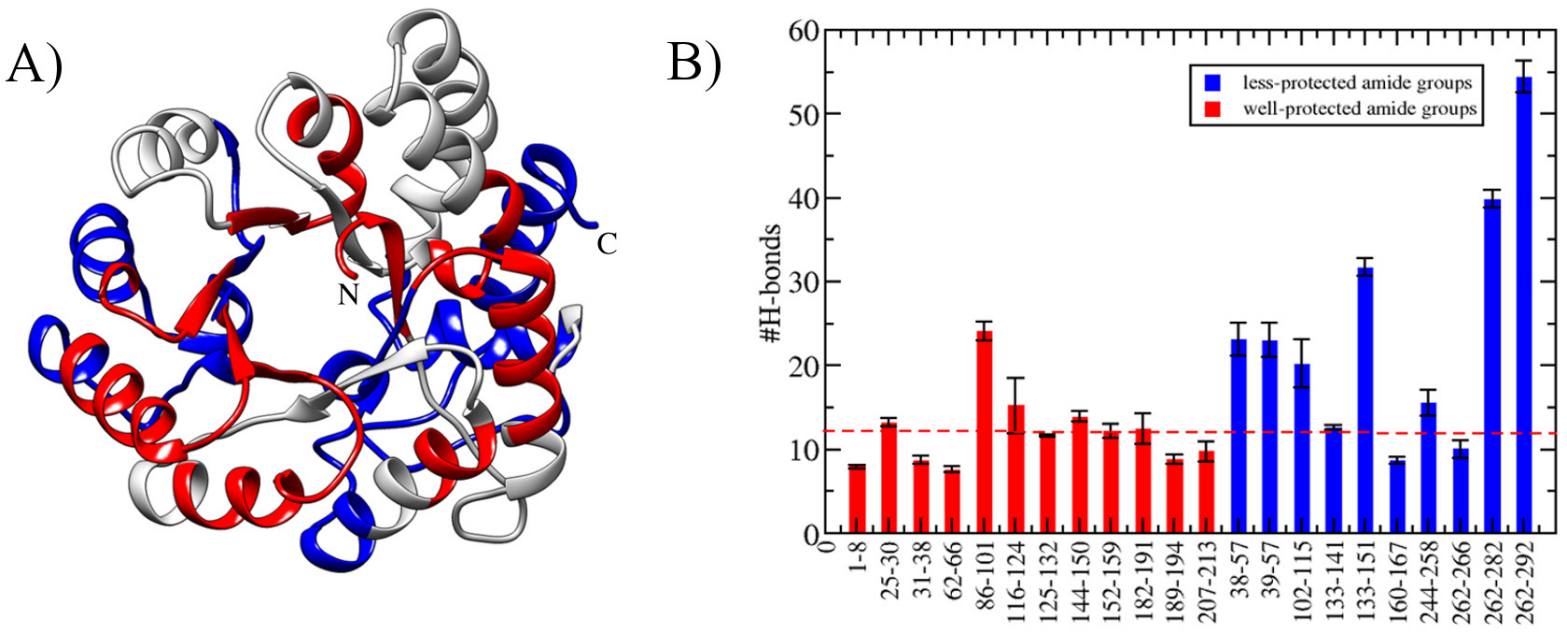
Experimental Validation of intermediate structures with HDX-MS data. A) Red and blue colored regions represents experimental characterization [51], displaying high- and less-protected amide groups, respectively. B) For all the peptide segments, number of hydrogen bonds with solvent molecules is shown. Dotted line indicates mean value of high-protected amide groups. The error bars denote the values computed from three 400 K trajectories populating I_2_ conformers.

Recent hydrogen-exchange coupled with mass spectrometry (HDX-MS) experiments have provided the most detailed information for folding intermediate of DapA [51]. HDX probes the exposure of a protein to D_2_O that induces rapid amide exchange in disordered regions that lack stable hydrogen-bonding. That is, if a particular region lacks stable hydrogen bonding, it will show less protection to amide groups and vice-versa. Therefore, HDX is an excellent technique for verifying rare states obtained from simulations by monitoring their H-bond pattern [52] as shown in previous studies [52, 53]. I_2_ conformers were analysed to compute protein-solvent hydrogen bonding for all experimentally characterised peptide segments as shown in Fig 3A and 3B. We found that H-bonding of more-protected amide groups show lesser number of protein-solvent hydrogen bonds (mean ≈ 12.15), while the less-protected regions show relative increase in hydrogen bonds (mean ≈ 23.89). This is in good agreement with experimental trend, where 8 out of 10 less-protected regions show higher protein-water H-bonds. For the sake of direct comparison, the fragment size was based on the experimental data [51]. Since our analysis depends on the number of hydrogen bonds, it might be correlated with the peptide size. Therefore, to explore this further, we normalised the number of hydrogen donors and acceptors in each peptide. In total, seven terminal residues (3–4 on each side) were choosen for all peptides in both protection groups. The peptides shorter than seven residues were not taken into account. Well-protected amide groups displayed on average ≈ 5.5 number of hydrogen bonds, in comparison to ≈ 11.67 for less-protected amide groups. Thus, the trend was consistent with our previous analysis using the full-length peptide and is independent of peptide length.

S5 Fig shows the atomistic view of these surface exposed amide regions in context of secondary structural elements. These segments constitute of residues 38–57 (*α*2, *β*2 and random coil), r102–115 (*β*4, *α*4 and random coil), r133–151 (*α*5 and random coil), r160–167 (*β*6 and random coil), r244–258 (*α*10), r262–292 (random coil and *α*11). While, most of the amide groups agree well with the experimental HX pattern, there are few discrepancies. Peptide segment 86–101, 116–124, 160–167, 262–266 show different trend for the simulations and experiments. In particular, 160–167 region belonging to TIM barrel core with low-protection is forming stable H-bonds in our simulations. On the other hand, r86–101 (*α*3) and r116–124 (*α*4) unfold completely and hence show higher number of H-bonds. The concordance between experimental observation and simulated results prompted us to further study the underlying structural characteristics of these intermediates with greater reliability.

### Structural characterization show disruption of TIM barrel topology and significant non-native *β*-sheet elements

**Fig 4:**
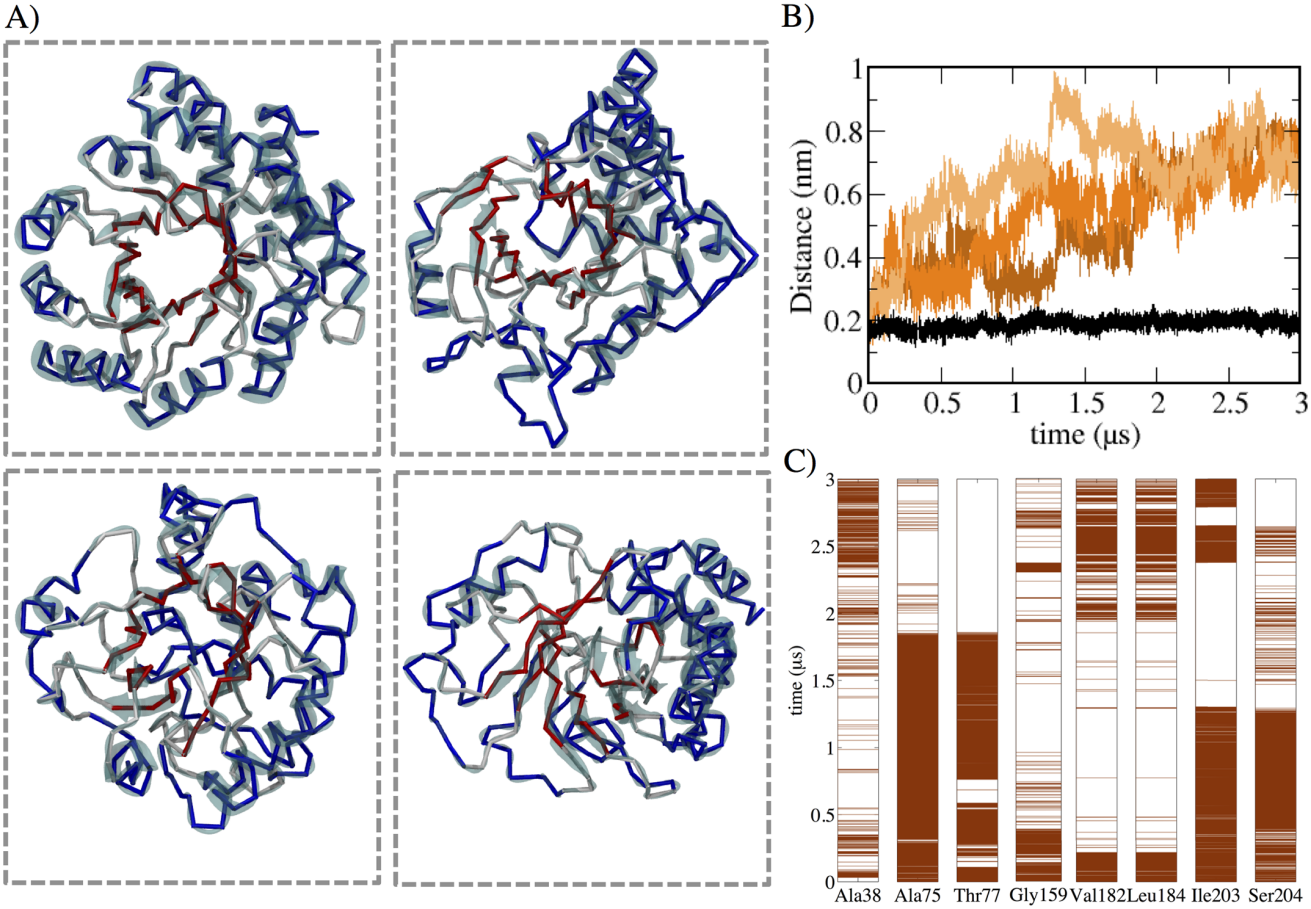
Conformational heterogeneity. A) Representative snapshots showing disruption of TIM barrel topology with *α* and *β*-region shown in blue and red color, respectively. B) Time evolution of distance between *α* and *β*-core for all three 400 K simulation shown in three shades of orange. For comparison, native 300 K is also shown in black. C) Time occurrence of representative *β*-core residues. The brown color represents the existence of beta-sheet secondary structure based on the dihedral angles.

The topology of the DapA monomer has a typical (*α/β*)_8_ TIM barrel fold with an additional C-terminal composed of three *α*-helices. This modular arrangement of repetitive *α*–*β* segments imparts structural stability to the TIM barrel fold [54]. Fig 4A shows the representative snapshots from constant temperature 400 K simulations, which shows large-scale conformational heterogeneity within DapA protein. The time occurrence of the distance between peripheral *α*-helices and *β*-sheet core shows an increase, indicating complete disruption of (*α/β*)_8_ TIM barrel topology (Fig 4B). In addition, the time evolution of the representative residues of many *β*-sheet showed fluctuations from *β* conformation to coil (Fig 4C). Further, these conformers also reveal a rather compact conformation. Previous studies have also shown that partially unfolded states possess molten globule geometry and native-like compactness [24, 55].

**Fig 5:**
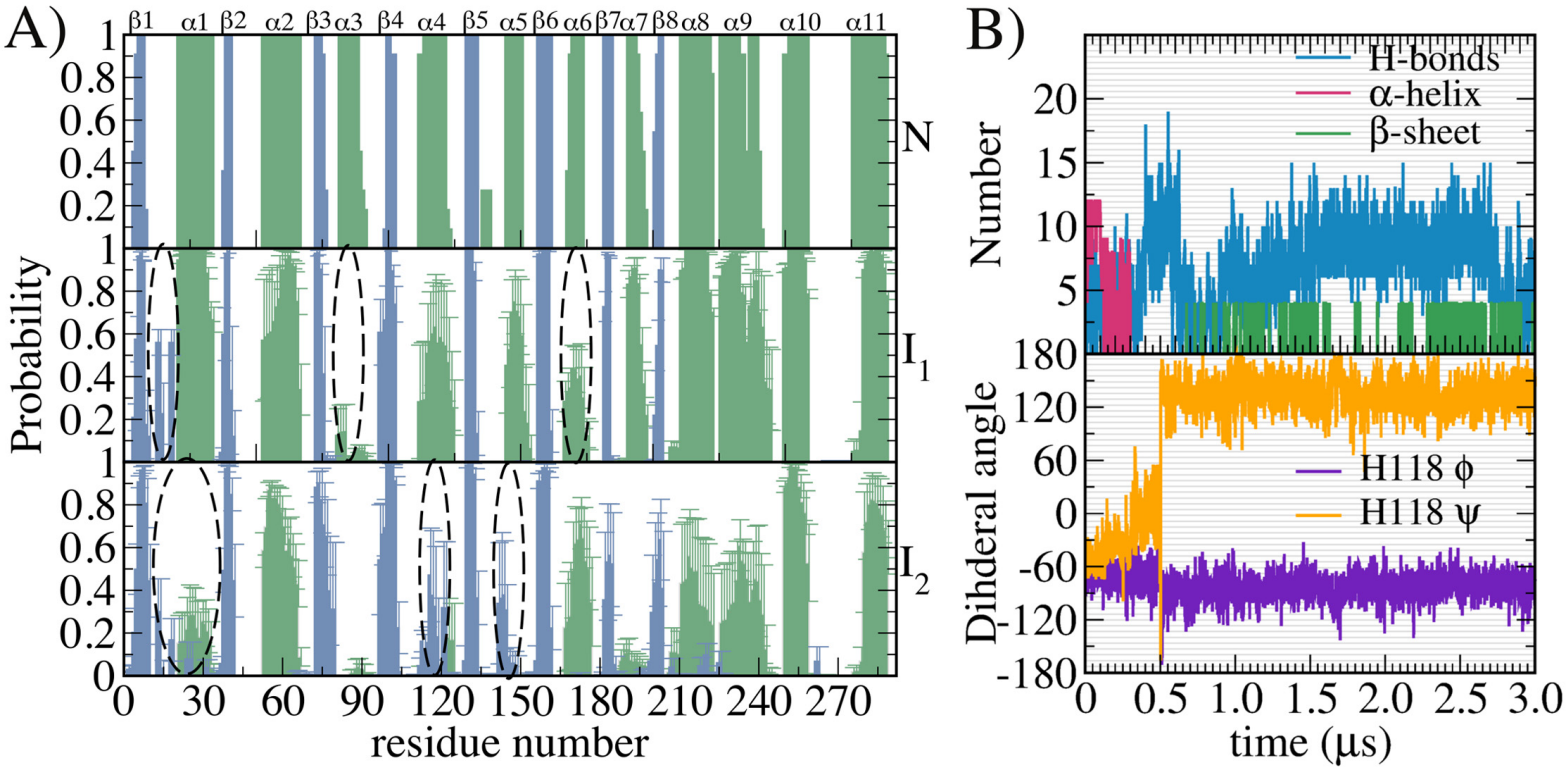
Structural characteristics of intermediates. A) Probability of each *α* and *β* secondary structural element normalized to the native protein structure as a function of amino acid residues is calculated. Comparison of I_1_ and I_2_ with N (native) reveals multiple conformational changes of *β*-sheets and *α*-helices as marked above in blue and green respectively. The secondary structures were assigned with DSSP. Error bars denote the standard deviation calculated from three simulations. B) The top panel shows the time occurrence of *α*-helix (magenta) to *β*-sheet (green) transition observed in *α*4 helix in one of the representative trajectory. In addition, *α*4 hydrogen bonds (in blue) and dihedral angle of His118 belonging to *α*4 (below) as a function of time are displayed, to indicate rearrangement of local bonding patterns.

To further probe the stability of these segments in the intermediates, we obtained the probability of secondary structure formation of native and intermediate ensembles by averaging over different conformations as a function of protein residues as depicted in Fig 5A. The peripheral *α*-helices show higher unfolding compared to inner core comprising of *β*-sheet. In comparison to equivalent secondary structure propensities of native topology, I_1_ and I_2_ showed 70% and 50% *α*–*β* content, respectively. Although native *β*-core remained more or less intact, there was an overall dramatic increase in non-native *β*-sheet formation as marked in Fig 5A. In I_1_, one of the prominent events was the unfolding of peripheral *α*3 (r81–93) along with partial structure loss of *α*4–6 (Fig 5A middle row). Surprisingly in the I_2_ state, two of these helices (*α*4–5) underwent conformational changes to form *β*-sheet structures (Fig 5A lower row). In addition, r11–19 belonging to the random coil region in the native DapA, transitioned to form a new *β*-sheet in the I_1_ and I_2_ states with 60% and 40% probability, respectively. Earlier studies have demonstrated that these *α* to *β* transition events in protein dynamics possess implicit role in protein aggregation [56, 57]. Although the potential energy of formation of *β*-sheet is higher than that of *α*-helix, the energetic balance is attributed to backbone entropy of sheets which is significantly larger to helices [58]. Fig 5B shows the kinetics of *α*-helix to *β*-sheet transition observed in *α*4 composed of r111–124 in one of the representative trajectory. After 300 ns, the *α*-helix underwent rearrangement of backbone hydrogen bonds to finally form stable *β*-sheet which persisted for several *μ*s. The time occurrence of dihedral angle of His118 was monitored and the change in angle clearly reflects the *α*–*β* conformational shift.

### Experiments and simulated conformers show substantial increase in surface-exposed hydrophobicity

**Fig 6:**
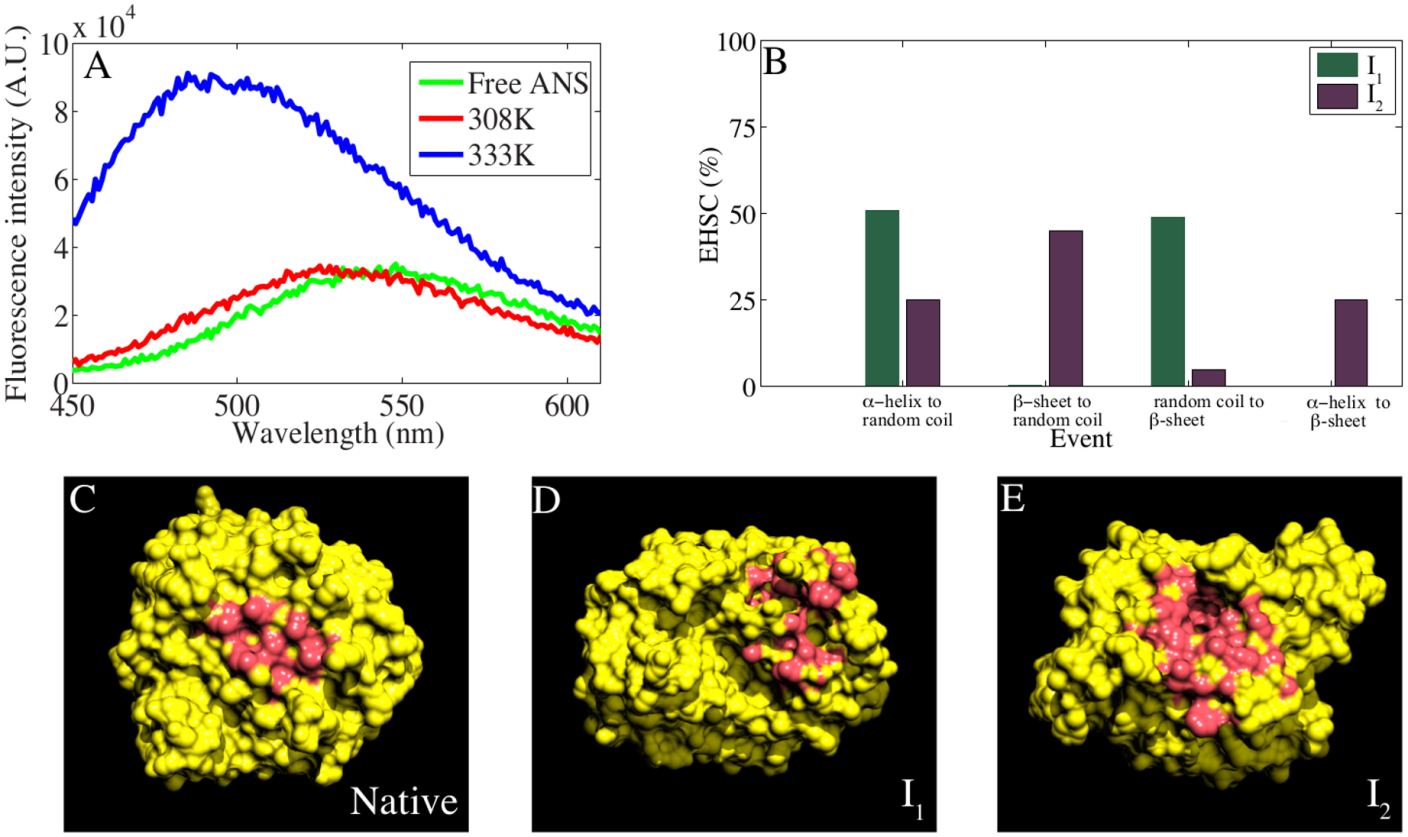
Surface exposed hydrophobic patches. A) Emission spectra of free 1–8 ANS (final 10 *μ*M) in buffer solution and bound to DapA incubated at 308 K and 333 K are plotted. The spectra was recorded after incubation with DapA (final 2 *μ*M) subjected to thermal denaturation by gradual increase in temperature. B) “Exposed Hydrophobic Surface Contribution” (EHSC) as a function of major conformational transition events in I_1_ and I_2_ are plotted. Four primary events contribute to conformational transitions in DapA, namely, i) *α*-helix to random coil ii) *β*-sheet to random coil iii) random coil to *β*-sheet, and iv) *α*-helix to *β*-sheet. The contribution of each event is attributed to the surface exposed hydrophobic patches i.e., how much percentage of *α*-helix to random coil event is giving rise to the total hydrophobicity in intermediate structures. C-E) Representative snapshots of N, I_1_ and I_2_ showing a clear increase in exposed hydrophobic patches in intermediates with respect to native, where exposed non-polar atoms of the contributing residues are highlighted in red color.

From our previous *in vitro* experiments with refolding intermediates, we found that the GroEL substrates possess molten globule like structures with varying degrees of secondary structure and exposed hydrophobic surface area [24]. We next asked the question whether surface hydrophobicity could explain specific structural attributes of the intermediates. To address this, we monitored the thermal unfolding-coupled increase in surface hydrophobicity of DapA by employing ANS (1-Anilino 8-sulphonic acid) as a probe. ANS is a widely used fluorescent probe which reports binding to exposed hydrophobic surfaces by increased fluorescence emission that shifts to lower wavelength, also known as “blue shift of ANS spectrum”. First we measured the fluorescence of the free probe which gave fluorescence emission at 530 nm wavelength. Upon binding the native protein at 25°C, the fluorescence spectrum remained unchanged as seen in Fig 6A. When we monitored ANS-binding to thermally unfolded DapA, formed by incubating the protein at 60°C, we observed a significant increase in fluorescence intensity and the emission peak shifted to 480 nm. The observed increase in the intensity is commonly attributed to hydrophobic clusters and unpacked side chains of the molten globule [59].

Further, to draw an atomistic view on varying levels of hydrophobic surface area, Fig 6C–6E displays the contiguous hydrophobic patches on the surface of representative native, I_1_ and I_2_ conformers, respectively, indicating the largest surface exposed hydrophobic patch in each structure. The complete list of patches in each ensemble is reported in S1 Table. The native topology’s largest patch (≈ 350 Å^2^) includes residues Phe2, Ile71, Pro72, Val98, Val182, as also shown in DapA crystal structure [60]. Few residues involved in dimer inter-facial region (r107–111) are also a part of exposed hydrophobic patches in native folded structure, implicating a greater role in binding sub-units of DapA. In comparison to native surface hydrophobicity (≈ 1400 Å^2^), we found significant increase in the degree of exposed surface area of I_1_ (≈ 2300 Å^2^) and I_2_ (≈ 2700 Å^2^) structures. In case of I_1_, the increase is associated with the N-terminal residues (r11–19) which formed a non-native *β*-sheet. This largest patch was further augmented with more interactions between these N-terminal residues and other helices (r47–48, r56–59, r246–250, r266–271). The partial unfolding of *α*2, *α*4–5 to random coil also contributed to the increase in the overall surface hydrophobicity of I_1_. The representative I_2_ structure exhibits major conformational changes, with top five patches displaying significant surface hydrophobicity (770 Å^2^, 580 Å^2^, 490 Å^2^, 420 Å^2^ and 350 Å^2^). The non-native *β*-sheet formation of *α*4 and *α*5 and, complete unfolding of *α*3 and *α*7 remarkably resulted in the overall increase of hydrophobicity in I_2_ structures. Due to the distorted *α*-helical periphery, part of *β*-barrel fold of DapA also contributed to the largest exposed patch in I_2_. In addition, we also observed the contribution from extended surface area of C-terminal helices (r258–275).

Since unfolding of DapA was directly correlated with an increase in hydrophobicity, we wanted to investigate prominent unfolding events responsible for this increase. From our simulations, we found that there were four events contributing to secondary structural transitions: the protein unfolds from either *α*-helix or *β*-sheet to form random coil; or the formation of *β*-strand from random coil or *α*-helix. We determined the contribution of these events as a function of exposed surface hydrophobicity as depicted in Fig 6B (see Methods for details). We found that the exposed hydrophobic surface area of I_1_ mainly arises from unfolding of peripheral *α*-helices and formation of non-native *β*-sheet elements. On the other hand, exposed hydrophobic surface area of I_2_ is originating from all four events, with native *β*-sheet to random coil being the prime contributor.

**Fig 7:**
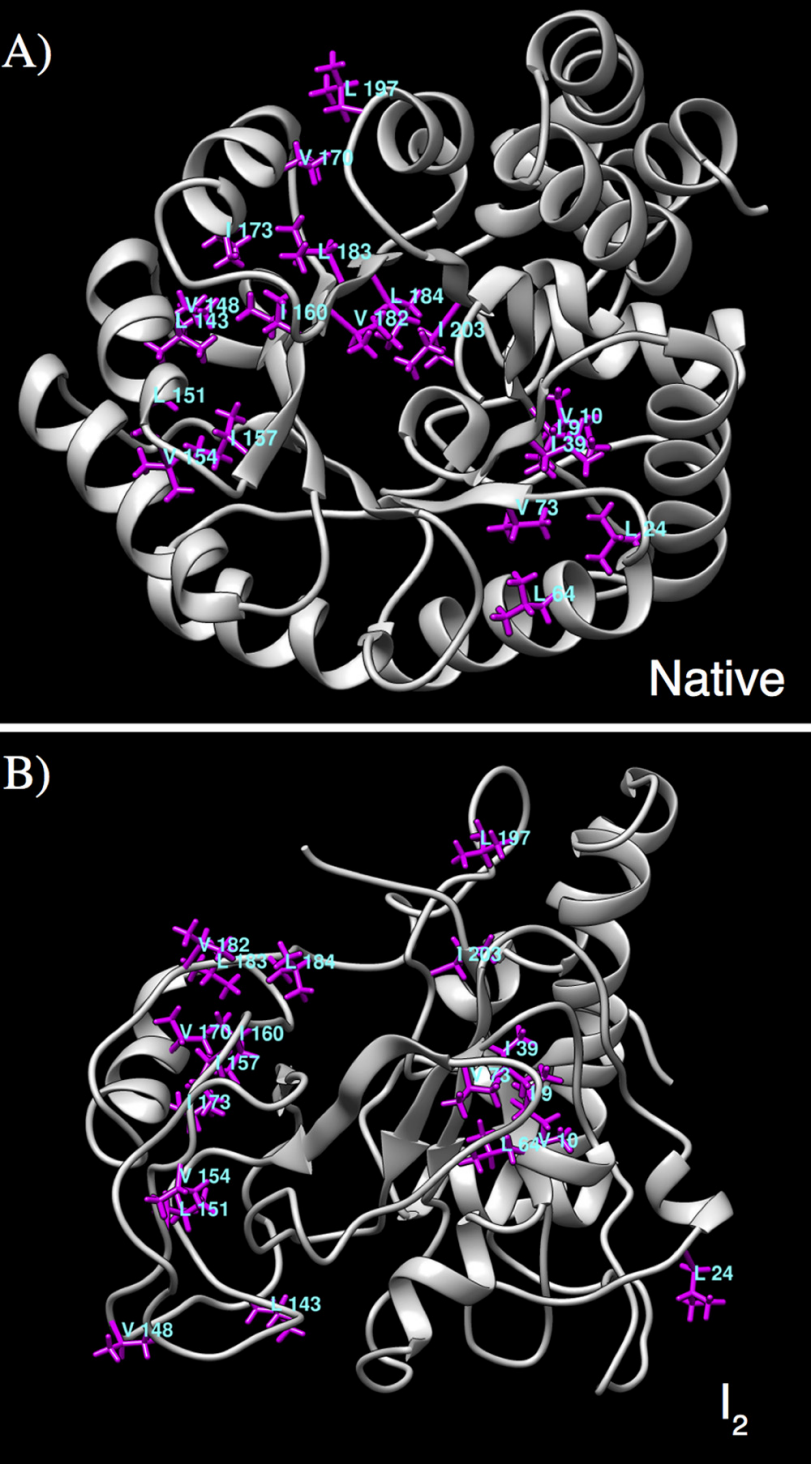
Surface exposed IVL clusters. Representative snapshot of the native (A) and the I_2_ structure (B), displaying IVL residues constituting a part of GroES-like binding motifs. In comparison to the native structure, these clusters are mostly solvent-exposed.

We also found that these hydrophobic patches are composed of Ile-Val-Leu rich clusters. In comparison with the native DapA (9 IVL residues), I_1_ had ≈ 17 exposed IVL residues and I_2_ conformers showed a significant increase with ≈ 27 exposed IVL residues. Interestingly, previous reports have suggested specific GroES-like binding motifs that are present in most of the natural GroEL-substrate proteins [61, 62]. In particular, they report seven plausible patterns in DapA, namely, G_IVL_G_A (1), G_IVL_G (2), and G_IVL (4) [61]. We traced these motifs in native and I_2_ conformers in our trajectories. Fig 7 displays the location of IVL residues constituting these motifs in the native (A) and the representative I_2_ (B) structure, clearly showing that these binding motifs are mostly buried in the former and are significantly exposed in the intermediate structure. Further, we calculated the average area per residue of all seven motifs and in comparison to the native (≈ 73.71 Å^2^), there is a substantial increase in the accessible surface area for intermediate structures (≈ 181.71 Å^2^). Thus, our results are consistent with previous observations that these motifs remain inaccessible in native state and become surface exposed in the unfolded conformations [61, 62].

### Analysis of non-native contacts in intermediates

**Fig 8:**
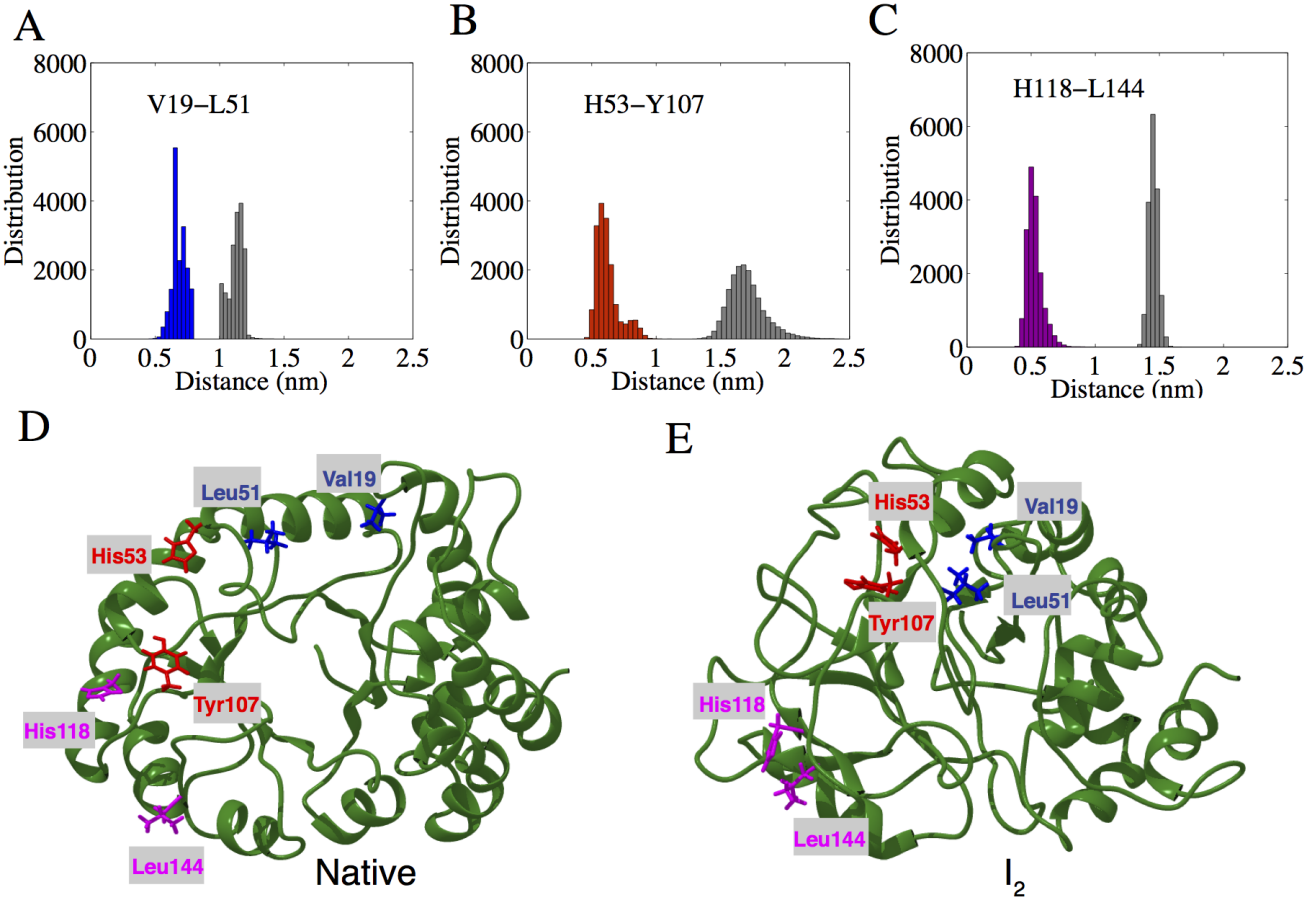
Non-native interactions. A-C) Distribution of C-*α* distances of three crucial non-native interactions in I_2_: Val191-Leu151, His53-Tyr107, and His118-Leu114, as shown in blue, red, and magenta, respectively. The native protein distance distribution is shown in gray, where these non-native contacts are largely absent. D-E) Representative snapshots of the native and I_2_ displaying the corresponding residues in colored stick representation.

Large proteins such as DapA whose folding is assisted by chaperones, the removal of non-native contacts is significant for discerning the factors that contribute to non-native structure stability. To address this quantitatively, we constructed inter-residue non-native contact map of DapA and identified crucial residues forming non-native interactions (S6 Fig). Fig 8A–8C shows the distance distribution of three key residue pairs, namely, Val19-Leu51, His53-Tyr107, and His118-Leu144, respectively. They were distantly apart in the native ensemble, but participated in persistent interactions within I_2_ ensemble as shown in representative snapshots of native and I_2_ structure (Fig 8D and 8E). In particular, Val19 underwent a conformational transition from random coil to non-native *β*-sheet in the both intermediates. As a result, it was exposed on the surface contributing to the overall hydrophobicity. Residue Tyr107 participates in the catalytic triad formation during DapA dimerization and is located on the interface I region of DapA [63]. In our simulations, we observed that this particular residue formed non-native interactions with His53 from *α*2, further resulting in partial exposure of the *β*-barrel in the I_2_ ensemble. Additionally, His118 and Leu144 belonging to *α*4 and 5, respectively also experienced partial unfolding in the intermediate conformers. Most of these non-native interactions contribute significantly to surface hydrophobicity, implying their greater role in stabilizing non-native conformers [64]. Previous MD simulations show that non-native contacts tend to make a notable contribution for collapsed or molten globule states along the unfolding pathways [53, 65].

### Energetics of GroEL-dependent and -independent substrate differs

**Fig 9:**
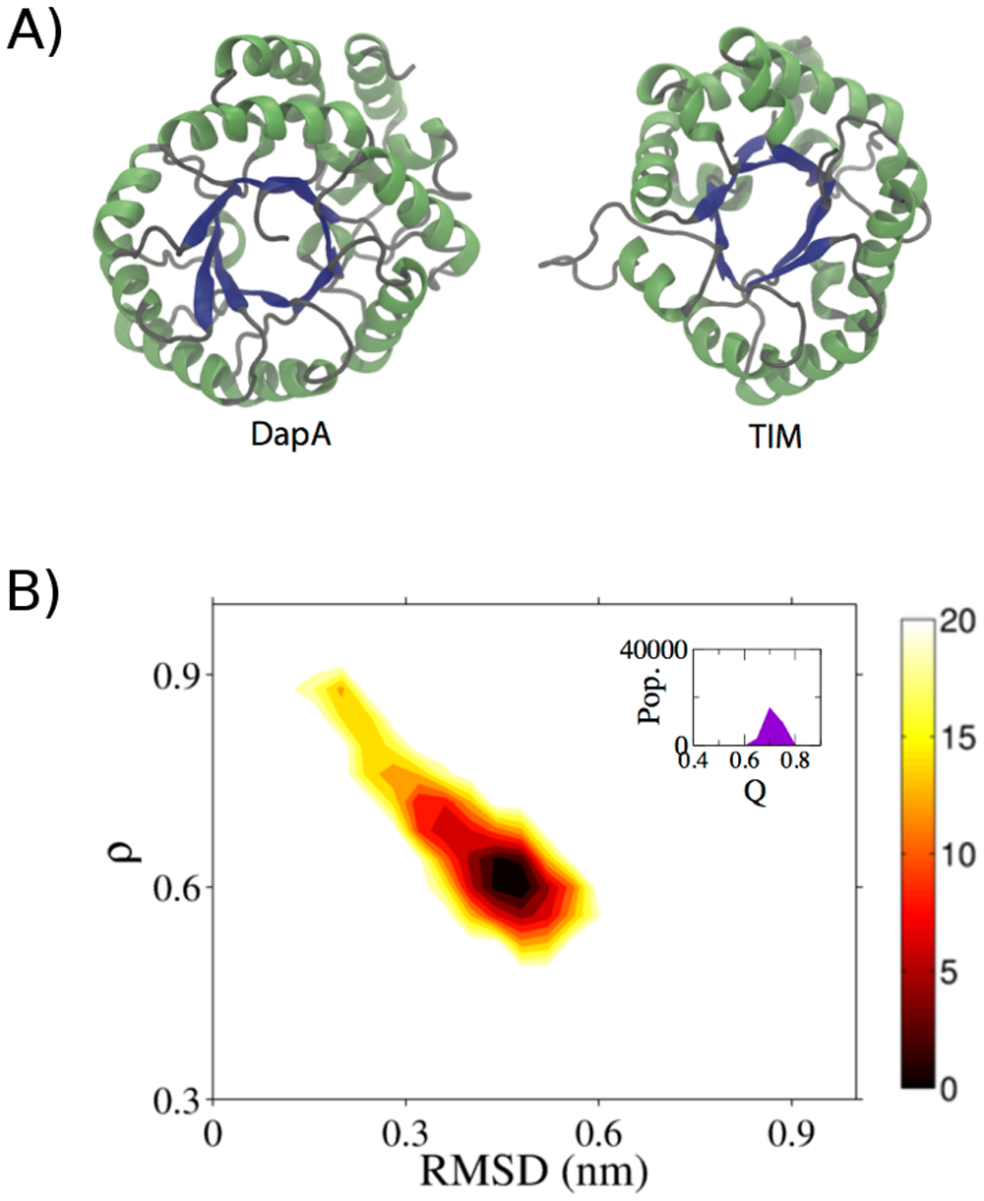
Energetics of GroEL-independent protein. A) Protein structures of DapA and TIM, highlighting their similar TIM barrel topology. B) Free energy contour map of TIM as a function of RMSD and *ρ* at 400 K as a control is depicted. The color bar denotes the Gibbs free energy in kJ/mol. The inset within the map shows distribution of fraction of native contacts, Q.

To understand whether a GroEL independent substrate follows a similar unfolding route, we performed additional MD simulations of a non-GroEL substrate, TIM protein as a control. Although both the proteins differ in chaperone specificity, they possess remarkable structural similarity in the form of typical TIM (*α/β*)_8_ topology as shown in Fig 9A. Surprisingly, compared to DapA free energy contour map (Fig 2C), TIM depicts a rather smooth unfolding showing minimal frustration with 70% native contacts intact as depicted in Fig 9B. The free energy barrier from the native state to the deep minimal bin is ≈ 6 kJ/mol. Structurally, the peripheral helices forming the TIM barrel were less flexible and degree of unfolding was dramatically reduced in TIM and the entire *β*-core remained completely intact. DapA showed the presence of a major (I_2_) and minor (I_1_) intermediate whereas TIM showed higher stability and complete absence of any intermediates. Earlier reports have indicated that the pathways and roughness can vary depending on geometry and length of the (*α/β*)_8_ of each TIM barrel [66]. While the comparison between DapA and TIM free energy surfaces provides interesting insights, folding/unfolding pathways of many such proteins will be required to generalise the observed trend.

### Quantifying residue interaction network

**Fig 10:**
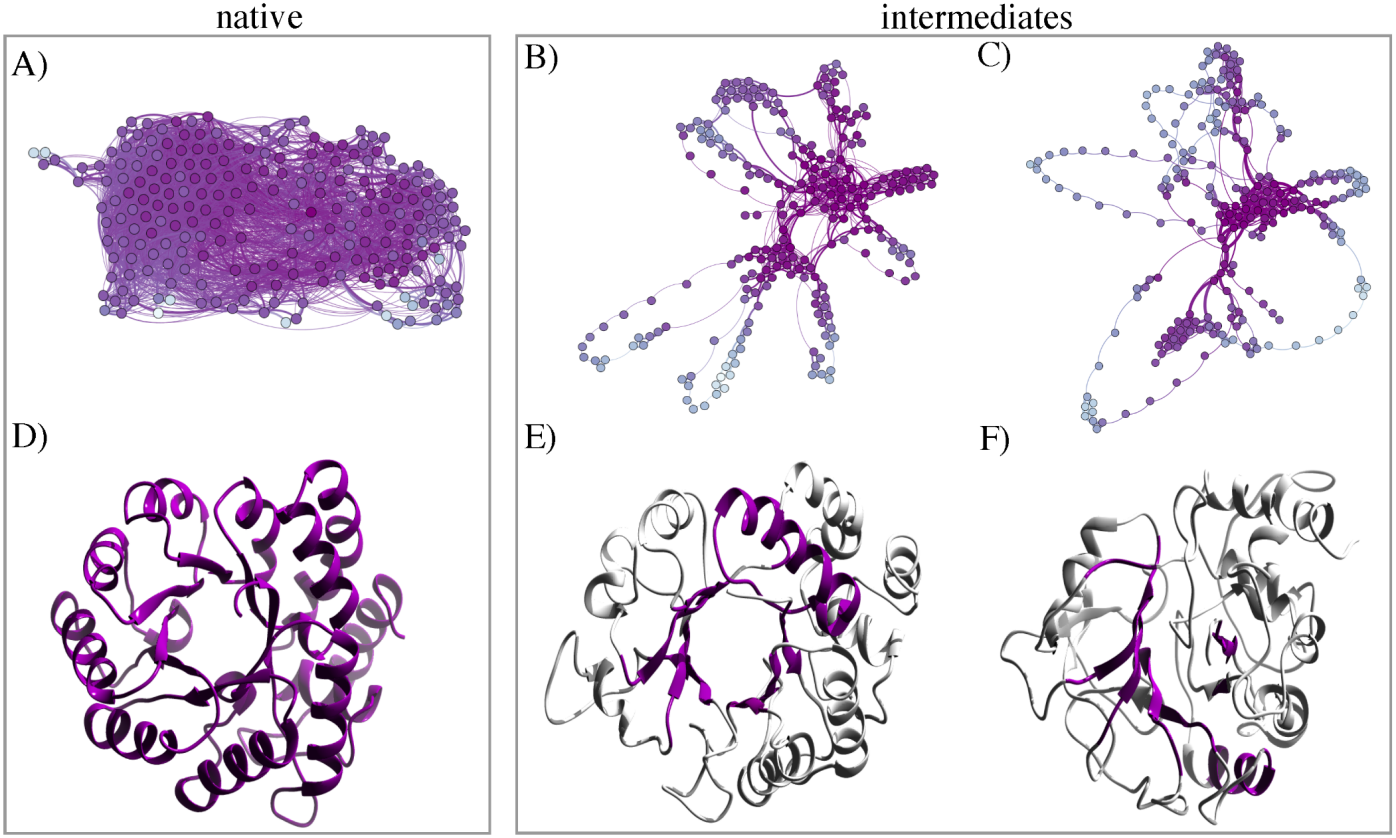
Intra-protein network representation of native and intermediate structures. Network graphs of N, I_1_, and I_2_ are shown in panel A-C, respectively. The nodes represents the amino-acid residues, communication pathways are depicted in bold lines and two connected residues by thin lines. Residues are colored from dark to light violet according to their communication efficiency, calculated by the number of residues to which they are connected. High communication between interacting residues describe pathways of well-defined interactions and such chains of residues constitute the communication pathway through which signals are transmitted efficiently. D-F) Snapshots of native and intermediates, highlighting the stable cluster within each structure. The 2D and 3D graphs are drawn with GEPHI and CHIMERA. The communication pathways are calculated using the MONETA tool.

Our analysis up to this point revealed two independent features of intermediate states; distinct structural characteristics, and significant increase in surface exposed hydrophobicity. However, the full impact of structural perturbations, is a combination of these two and many more. Thus, to discern global dynamic changes within unfolding intermediates, we used a network approach to analyze the intra-protein communication pathways and construct graphs that are based on geometry of protein conformations. We built the protein network that explores a) the intra-molecular long-range interactions, and b) dynamical correlations computed from MD trajectories to identify highly connected nodes, also known as hubs (see Methods section for detailed protocol). Similar methodology has also been computed to understand allosteric communication between native and mutated KIT Receptor Tyrosine Kinase [39]. Fig 10A–10C shows the N, I_1_, and I_2_ network graphs along with the corresponding three-dimensional representation of each structure shown below (Fig 10E and 10F). Here, amino acids are denoted by nodes which are linked by edges representing connections of different nature such as peptide bonds, non-covalent interactions etc. The architecture of the network topology also reveals communication pathways (dark bold lines) which are formed by either highly or moderately communicating residues.

Strikingly, in contrast to intricate well-knit native topology, intermediate graphs show dramatic decrease of connected nodes, indicating the absence of large number of long-range interactions. In strong contrast to random networks, we obtained distinct architecture underlying intermediates in the form of stable hubs, primarily contributed from *β*-sheet residues (for zoomed images, see S7 Fig). In I_1_ the two stable clusters majorly correspond to the native *β*-sheet along with *α*8, imparting conformational stability to the overall structure. The distant connections (light-colored nodes/edges) depict the initial unfolding of *α*3–6 resulting in the extended arrangement of these segments. Considering the fact that I_2_ ensemble experienced major structural fluctuations, part of core *β*-sheet (*β*3–6) still contributed to the main dense cluster of nodes. The global shape of the derived network highlights the loosely connected peripheral *α*-helices which were mostly unfolded. The substantial exposure of the core *β*-sheet is significantly evident as compared to central pattern of the native and I_1_ network graphs. The second cluster encompasses moderately connected *α*2 (r52–68), participating in the communication pathway linked to the stable *β*-strands. Together the networks discussed above provide a comprehensive description of unfolded DapA behavior, capturing local dynamics, propagation of signals to distal nodes and the global response of the protein structure to perturbations. Yet, they also illustrate the rather diverse dynamical states a protein can generate in an unfolded ensemble. Network approaches on native geometry of many proteins showcase high average connectivity of residues in *α*/*β* protein class [67]. The protein structural graphs are reported to form small world networks i.e., node’s edges lead to neighboring residues (close in primary sequence) but also comprising of long-distance connections to residues which are far apart. This characteristic intricate feature is valid for most native globular proteins [68–70]. In the present work, the network approach thus provides fascinating insights into the topology and dynamics of complex systems such as protein folding intermediates.

### Discussion

This work addresses longstanding question of whether structural scaffolds exist in unfolded proteins, that are targeted to chaperone. Historically, molecular recognition of chaperone-targeting proteins has remained largely enigmatic [10, 25]. Several challenges impede our understanding of chaperone binding to partially folded/unfolded proteins. These proteins can associate non-specifically and therefore, have an inherent propensity to aggregate. To add the complexity, unfolded proteins en route to chaperone binding are highly dynamic in nature, and therefore conformational transitions are experimentally difficult to probe. In recent years, characterization of non-native substrates bound/unbound to GroEL is possible due to significant technological advances by means of HDX-mass spectrometry [51], NMR [19], and electron microscopy [71–73]. Thus, novel insights emerge from these exciting studies about complex binding, however, several questions remain: the detailed atomistic understanding of unfolded proteins, differences with respect to native structure, what are the factors that contribute to these differences (non-native, if any), and finally how these local and global perturbations of unfolded protein dynamics may influence chaperone binding. On the other hand, computer simulations fruitfully combined with experiments are now emerging as powerful tools to reveal molecular details of conformational dynamics of unfolded/partially-folded structures [74].

Here, we have characterized the populated intermediates of an obligate GroEL substrate, DapA using explicit-solvent high-temperature unfolding simulations i.e., starting from the folded structure. Unfolding simulations provide several leverages as they proceed from an experimentally well-characterized structure, and more importantly for larger globular proteins one can delineate the details of intermediate structures and their dynamic transitions [41, 45]. Recently, Shaw et al. showed that the unfolded state of ACBP is in close agreement with the NMR experiments, and the simulations capture crucial aspects of local and global structure [75]. The characterization of our unfolding simulations is based on the principle of microscopic reversibility, which implies that intermediates for folding and unfolding are the same [45]. Thus, unfolding simulations provides a powerful platform for studying denatured proteins [75, 76]. However, obtaining statistically reliable rare states is computationally intensive and requires an integrated computational-experimental approach [77].

In this work, we were particularly interested to capture robust intermediate states of DapA folding, as we have previously reported that these are crucial for chaperone targeting [24]. Free energy contour maps revealed two major basins with highly populated I_1_ and I_2_ states. We found that intermediate structures show residual amount of secondary structure, with fraction of native contacts (Q) ranging from 65% (I_1_) to 40% (I_2_). For a relatively large system (such as DapA; 292 residues), a complete characterization of free energy landscape, involving mapping of free energies of all non-native structures and the native state is extremely challenging. Therefore, sampling in the vicinity of an ensemble of folding or unfolding pathways projected onto two-dimensional reaction co-ordinate maps, provides key insights into the relevant conformational space [78].

We also confirm the existence of intermediate ensemble using REMD simulations. However, it is important to note that, the REMD simulations were used as validation for intermediate conformers obtained by CTMD simulations. In this study, the time per replica was 700 ns, which translates into 67.7 *μ*s of MD (96 replicas each) simulation time. Although, this represents the most extensive REMD study for a large protein to date, we found that our conventional MD simulations (27 *μ*s) show better conformational sampling at higher temperature. In an earlier study, folding of of a small protein HP36 showed multiple folding events using multicanonical replica-exchange simulations that utilizes multicanonical algorithm and replica-exchange method [79].

This observation supports the notion that the denatured globular proteins possess considerable degree of secondary structure and is compact in nature [76]. Next, determination of the structural characteristics of intermediate ensemble shows complete unfolding of many peripheral helices which further propagates to the beta barrel core leading to its exposure. Surprisingly, we observed the formation of non-native *β*-sheets originating from either alpha helices or random coil region. Kinetic monitoring of one such representative *α* to *β* transitions involve complete re-arrangement of backbone hydrogen bonds (see Fig 5). Previous report highlights an important mechanistic insight into these transitions and suggests that metastable *β*-hairpin intermediate states provide a missing link in understanding amyloidogenesis [58].

In general, substrate promiscuity for GroEL chaperonin is an intriguing matter as GroEL-assisted folding of proteins involve broad substrate specificity. Several reports suggest the role of hydrophobic interactions as the driving force for the interaction of substrates and GroEL [11]. Although, it is known that the presence of exposed non-polar surfaces of unfolded/partially folded substrates bind with GroEL [17, 21, 80–82], our understanding of atomistic insights of unfolded structures is limited. Structural and dynamical characterization of non-polar contiguous surfaces of DapA I_1_ and I_2_ states revealed the presence of large surface exposed hydrophobic patches. The secondary structural composition of these patches highlighted the contribution of non-native *β*-sheet elements, exposed native *β*-core and unfolded peripheral helices. These observation relied on the synergy between computer simulations and experiments, both addressing conformations with increase in surface exposed hydrophobic patches. ANS binding experiment showed the presence of large hydrophobic surfaces on *in vitro* unfolding intermediates. These findings agree with previous observations that the presence of large accessible hydrophobic patches possibly drives GroEL interaction [17, 21, 82]. Furthermore, the presence of characteristic hydrophobic IVL clusters on the surface of non-native intermediates has been shown as an important criteria for GroEL recognition [61, 62]. We traced GroES-like binding patterns on GroEL substrates during DapA unfolding simulations. We observed that the IVL residues constituting these patterns became significantly exposed in the characterised intermediates with respect to native conformations where they remained majorly buried.

The TIM barrel fold has been proposed as the preferred folding topology for the GroEL substrates [7, 9]. Thermal unfolding of TIM, a TIM barrel domain protein independent of GroEL assisted folding illustrates an ensemble of a rather homogeneous conformational states in our simulations. In contrast to DapA, TIM displays higher thermo-stability during unfolding whereas DapA, a GroEL dependent folder populates metastable intermediate states. Similar comparison between DapA and NanA (GroEL-independent) folding was also reported, where both proteins undergo segmental formation but show differences in their folding pathways [51]. Previous studies also indicate that GroEL substrates have lower folding propensities compared with GroEL-independent proteins [13, 83]. Raineri et al. found that GroEL substrates are less hydrophobic than GroEL-independent proteins and also suggested that they are more conserved than non-substrates [23]. However, there exists several TIM barrel like proteins which are dependent on the GroEL and several that are not, folding/unfolding pathways of many such proteins will be required to satisfy a general trend.

In recent times, computer simulations can capture higher time-scales and thus achieve an effective overlap with several experiments [84]. Here, the characterized unfolding intermediates have been substantiated by the hydrogen/deuterium exchange and mass spectrometry experiments [51]. Recently, all-atom simulations were benchmarked using hydrogen exchange experiments and provides a general framework to experimentally validate folding simulations [52]. Additionally, existence of signature networks underlying DapA intermediate structures may play an active role in the chaperone-substrate complex formation. The usage of structure-based topological maps in protein dynamics and signal propagation has been extensively studied before [85–87]. Interestingly, topological properties of CI2 and C-Src SH3 protein conformations reveal that the network topology changes toward a specific one when protein crosses the transition barrier [86]. The present simulations predict the plausible intermediate state and may now open the door to investigate detailed study of chaperone-substrate binding. Large-scale identification of these hot-spots (central hubs) within unfolded states that target chaperone, will greatly enhance our mechanistic understanding of several misfolding diseases.

## Supporting Information

S1 Table List of significant hydrophobic patches. Exposed surface hydrophobic patches on Native, I_1_ and I_2_ structures with surface area ≥ 300 Å^2^ are reported.
(PDF)

S1 Fig Additional Two-dimensional Maps. Free energy contour maps of DapA as a function of RMSD-Q and RMSD-SASA for three different temperatures, namely; A-B) 310 K, C-D) 360 K and E-F) 400 K, respectively. The color bar denotes the Gibbs free energy in kJ/mol.
(TIF)

S2 Fig Structural comparison of constant-temperature and replica-exchange molecular dynamics simulations. Representative snapshots of the intermediate structures derived from 400 K of replica-exchange (A) and constant-temperature simulations (B). Conserved to variable regions are coloured ranging from blue to red, representative of the B-factor values derived from C-*α* RMSF analysis. Structures derived from both simulations were found to be similar and additionally structural fluctuations (seen above) are also localised to same regions.
(TIF)

S3 Fig Convergence of Simulations. Time history of I_2_ conformation sampled at least once in (A) replica-exchange and (B) constant-temperature molecular dynamics simulations For the latter, intermediate conformations derived from free energy surface was mapped onto time course of three constant temperature simulations The occurrence of the rare metastable state was observed at least once by 1.5 *μ*s across all simulations. For probing the stability, we extended the simulations until 3 *μ*s. Similar protocol was applied to replicas obtained from REMD and the stability was observed around 200 ns.
(TIF)

S4 Fig Comparison between REMD and CTMD Simulations. Presence of intra-molecular mainchain hydrogen bonds as a function of different temperatures in REMD and CTMD simulations are shown in Panel A and B, respectively The box plot representation is shown with central red mark representing median, the edges of the box are the 25th and 75 percentiles. The black points are the whiskers which represents the extent of data points and plus signs (in red) are the outliers of each column.
(TIFF)

S5 Fig Experimental Validation. Surface representation of I_2_ structure displaying surface exposed low- amide protected peptides in green as derived from recent DapA refolding experiments (Georgescauld et. al., 2014).
(TIF)

S6 Fig Non-native contact map of I_2_ intermediate. Non-native interactions were analyzed within 7 Å with respect to native topology. Black and red squares represent native and I_2_ average topology respectively. Further, significant and persistent interactions were calculated using g_dist and are plotted in Fig 5.
(TIF)

S7 Fig Snapshots of densely clustered hubs in the intra-protein network of the two intermediates. I_1_ panel showing clusters of A) *β*1–3,8 *α*8 and B) *β*4–7. I_2_ panel showing a dense cluster of C) *β*3–6.
(TIF)
