# Supplementary material for: Decoding Structural Properties of a Partially Unfolded Protein Substrate: En Route to Chaperone Binding
Source: PLoS Comput Biol. 2015 Sep 22;11(9):e1004496. doi: 10.1371/journal.pcbi.1004496 (PMC4578939; doi:10.1371/journal.pcbi.1004496)
Supplement: S1 Table — Exposed surface hydrophobic patches on Native, I1 and I2 structures with surface area ≥ 300 Å2 are reported. (PDF) [file pcbi.1004496.s001.pdf]

S1 Table: List of significant hydrophobic patches

Structure	Number of patches	Residue contribution	Patch size (Å^2)
Native	1	1, 2, 3, 71, 72, 98, 129, 155, 156, 158, 182	350
	2	44, 47, 49, 50, 138, 246, 247, 249, 250, 270, 271	313
I_1	1	11, 13, 15, 16, 17, 19, 44, 47, 48, 56, 58, 59, 244, 246,247, 248, 249, 250, 254, 266, 267, 268, 269, 271	830
	2	107, 108, 109, 110, 111, 112, 113, 140, 142, 144, 145, 146	487
	3	245, 259, 278, 280, 281, 283, 284, 287, 292	418
	4	43, 49, 50, 51, 101, 103, 104, 133, 135, 136, 137, 138, 163, 164, 166	355
I_2	1	2, 4, 6, 37, 38, 70, 71, 72, 74, 90, 92, 93, 94, 96, 97, 98, 126, 127, 128, 129, 131, 156, 158, 182, 184, 202	770
	2	258, 259, 260, 262, 264, 265, 267, 269, 270, 271, 272, 273, 274, 275	580
	3	187, 189, 190, 192, 193, 194, 195, 196, 223, 224, 226, 227, 234, 237, 238, 240	490
	4	106, 107, 108, 139, 140, 143, 144, 145, 146, 166, 168, 169, 172	420
	5	53, 84, 87, 88, 89, 115, 116, 117, 118, 119	350
