# Supplementary figures and images for: Decoding Structural Properties of a Partially Unfolded Protein Substrate: En Route to Chaperone Binding
Source: PLoS Comput Biol. 2015 Sep 22;11(9):e1004496. doi: 10.1371/journal.pcbi.1004496 (PMC4578939; doi:10.1371/journal.pcbi.1004496)

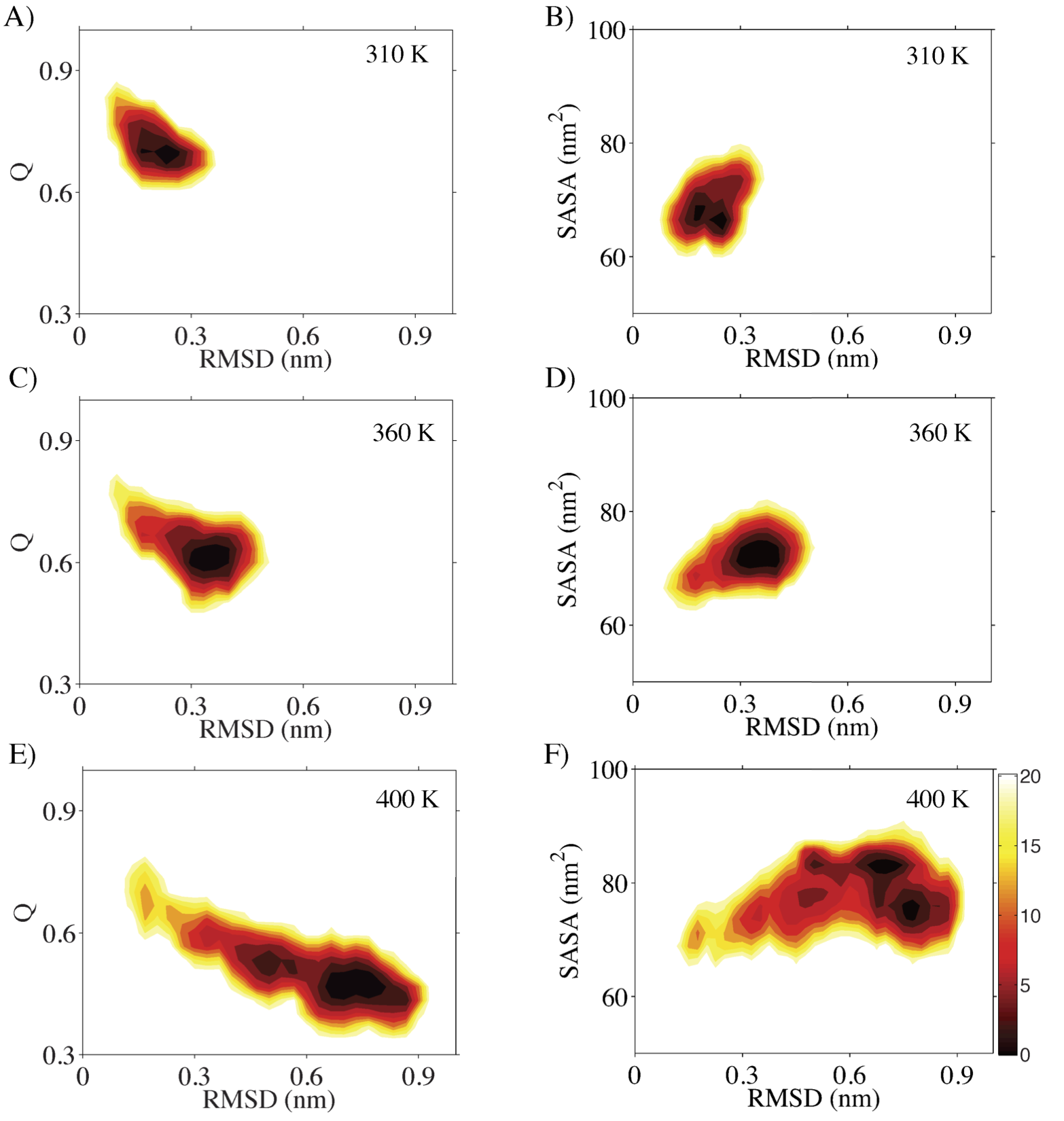

Supplement: S1 Fig — Free energy contour maps of DapA as a function of RMSD-Q and RMSD-SASA for three different temperatures, namely; A-B) 310 K, C-D) 360 K and E-F) 400 K, respectively. The color bar denotes the Gibbs free energy in kJ/mol. (TIF) [file pcbi.1004496.s002.tif]

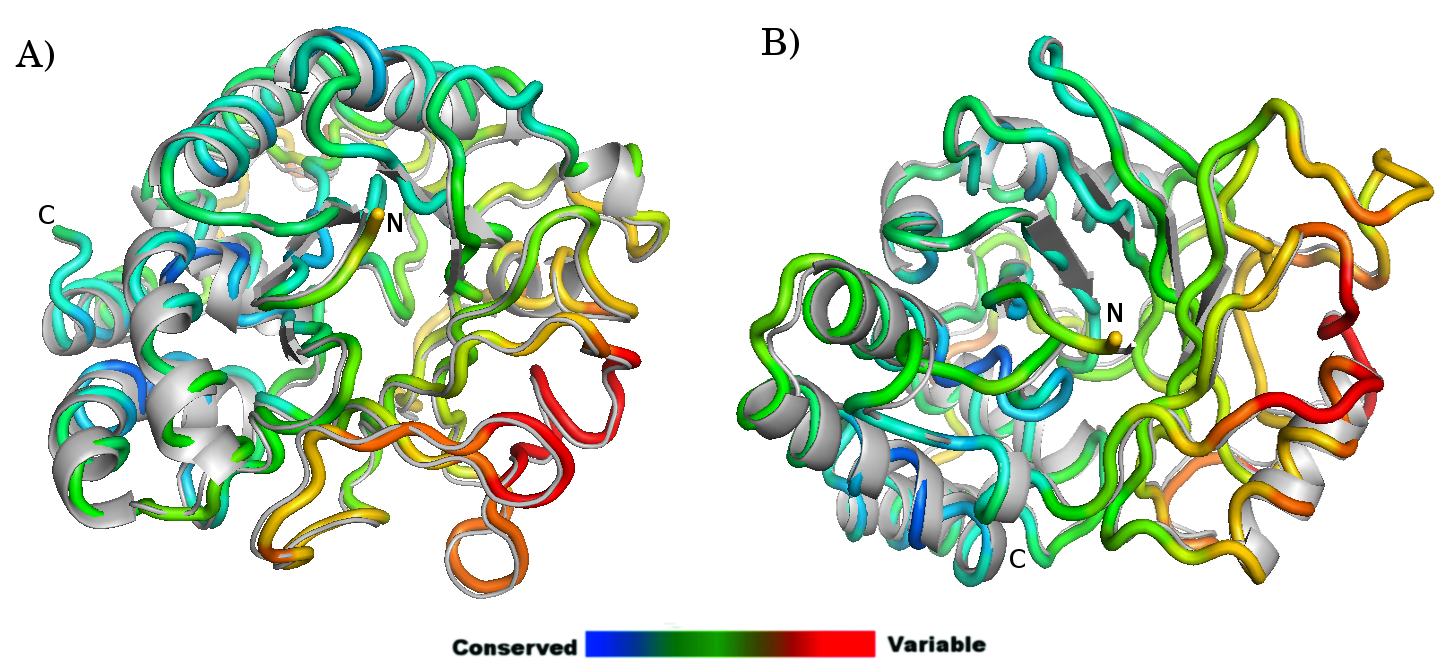

Supplement: S2 Fig — Representative snapshots of the intermediate structures derived from 400 K of replica-exchange (A) and constant-temperature simulations (B). Conserved to variable regions are coloured ranging from blue to red, representative of the B-factor values derived from C-α RMSF analysis. Structures derived from both simulations were found to be similar and additionally structural fluctuations (seen above) are also localised to same regions. (TIF) [file pcbi.1004496.s003.tif]

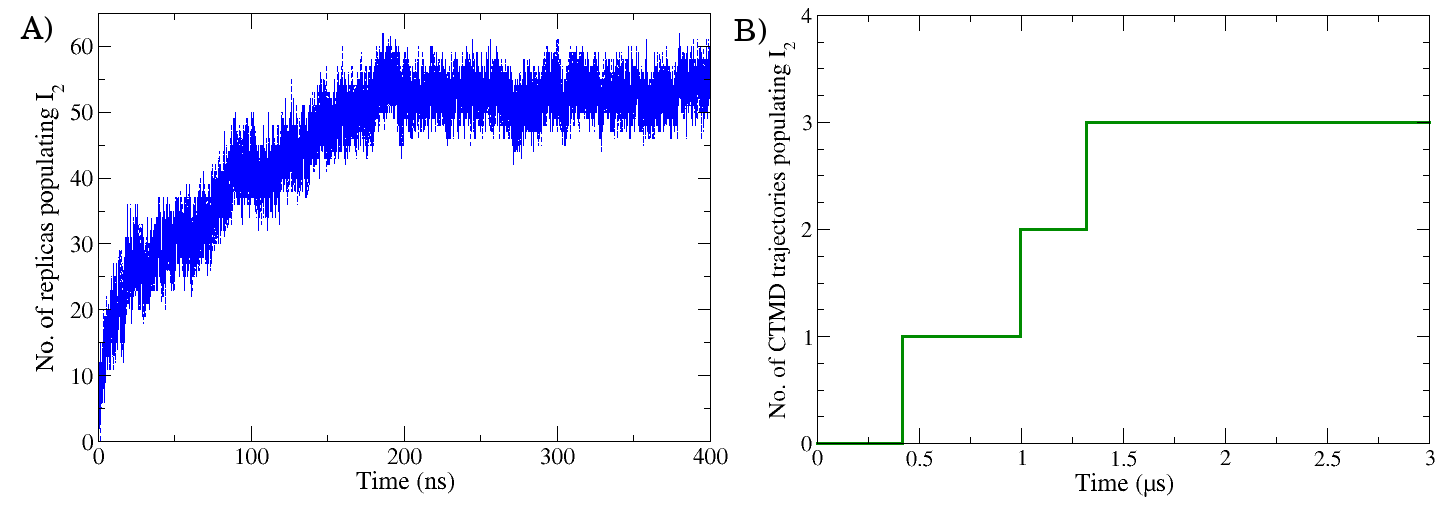

Supplement: S3 Fig — Time history of I2 conformation sampled at least once in (A) replica-exchange and (B) constant-temperature molecular dynamics simulations For the latter, intermediate conformations derived from free energy surface was mapped onto time course of three constant temperature simulations The occurrence of the rare metastable state was observed at least once by 1.5 μs across all simulations. For probing the stability, we extended the simulations until 3 μs. Similar protocol was applied to replicas obtained from REMD and the stability was observed around 200 ns. (TIF) [file pcbi.1004496.s004.tif]

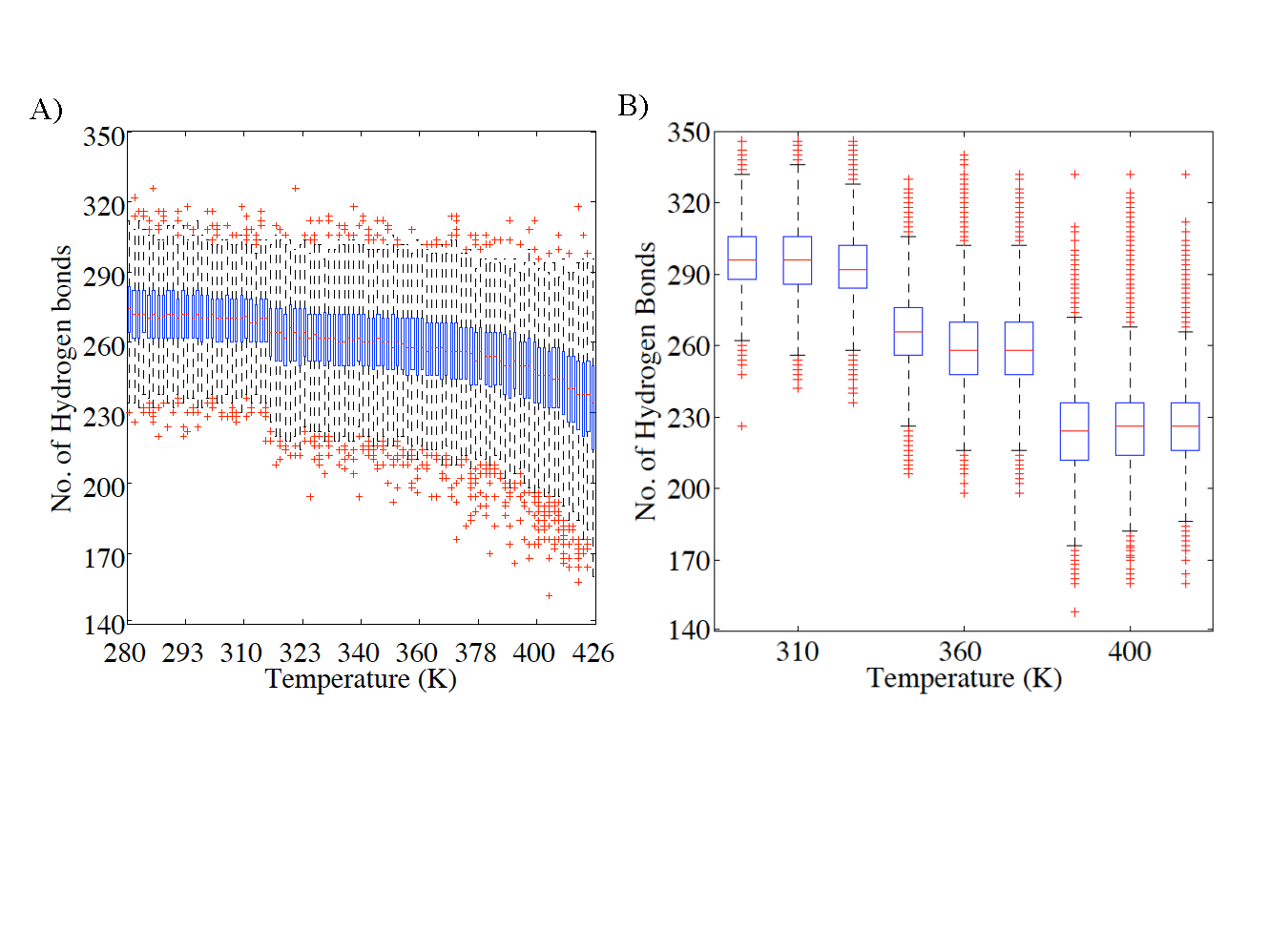

Supplement: S4 Fig — Presence of intra-molecular mainchain hydrogen bonds as a function of different temperatures in REMD and CTMD simulations are shown in Panel A and B, respectively The box plot representation is shown with central red mark representing median, the edges of the box are the 25th and 75 percentiles. The black points are the whiskers which represents the extent of data points and plus signs (in red) are the outliers of each column. (TIFF) [file pcbi.1004496.s005.tiff]

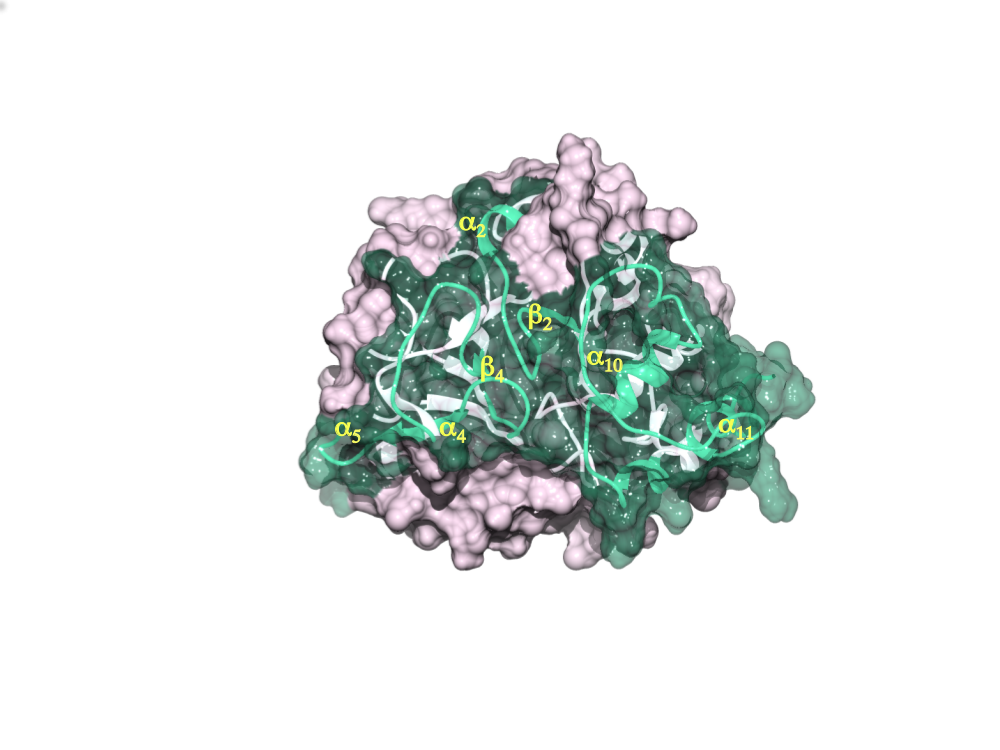

Supplement: S5 Fig — Surface representation of I2 structure displaying surface exposed low- amide protected peptides in green as derived from recent DapA refolding experiments (Georgescauld et. al., 2014). (TIF) [file pcbi.1004496.s006.tif]

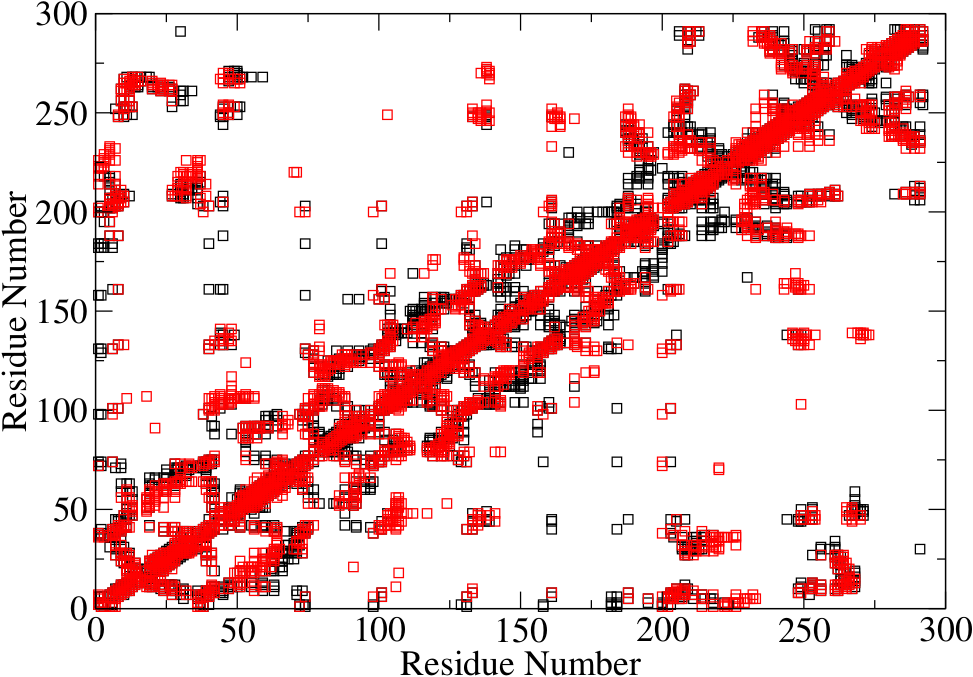

Supplement: S6 Fig — Non-native interactions were analyzed within 7 Å with respect to native topology. Black and red squares represent native and I2 average topology respectively. Further, significant and persistent interactions were calculated using g_dist and are plotted in Fig 5. (TIF) [file pcbi.1004496.s007.tif]

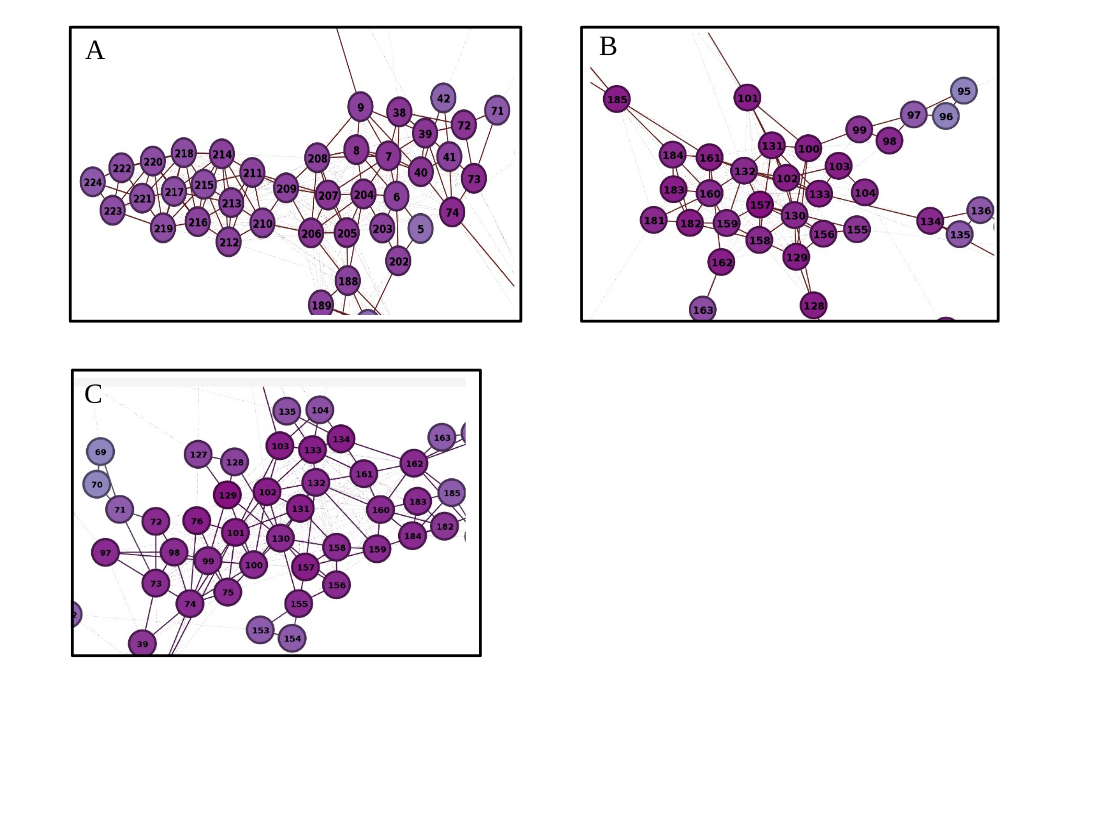

Supplement: S7 Fig — I1 panel showing clusters of A) β1–3,8 α8 and B) β4–7. I2 panel showing a dense cluster of C) β3–6. (TIF) [file pcbi.1004496.s008.tif]
